# Unveiling DNAJB12 and DNAJB14 as crucial chaperones in hepatitis B and D virus particle morphogenesis

**DOI:** 10.1016/j.isci.2025.113897

**Published:** 2025-10-30

**Authors:** Léna Angelo, Richard Boulon, Patrick Labonté

**Affiliations:** 1 INRS-Centre Armand-Frappier Santé Biotechnologie, Laval, QC H7V 1B7, Canada

**Keywords:** Natural sciences, Biological sciences, Microbiology, Virology

## Abstract

During chronic HBV infection, the massive secretion of HBV envelope proteins (HBsAg) as non-infectious subviral particles (SVPs) remains a significant challenge in achieving a functional cure. Despite this, the HBsAg folding process, essential for HBV and HDV particle morphogenesis, remains poorly understood. DNAJB12 and DNAJB14 are two recently identified co-chaperones implicated in transmembrane protein folding. Utilizing the nucleic acid polymer REP 2139 as a bait, we identified DNAJB12 as a REP 2139 interactor, and its knockdown impedes the morphogenesis and secretion of SVP and HBV virions. Conversely, DNAJB14, which did not interact with REP 2139, selectively impaired the morphogenesis of virions. Additionally, knockdowns of DNAJB12 and DNAJB14 hindered the production of infectious HDV. As DNAJB12 knockdown recapitulated REP 2139 antiviral effects observed in clinical trials, our findings highlight DNAJB12 as the potential primary target of REP 2139 and uncover functional roles for DNAJB12 and DNAJB14 in HBV and HDV life cycles.

## Highlights

• DNAJB12 is crucial for the morphogenesis of HBV Dane and SVPs as well as HDV virions
• DNAJB14 does not affect HBV spherical SVPs but is required for HBV and HDV formation
• DNAJB12, but not DNAJB14, is targeted by REP 2139 via its domain of unknown function

## Graphical abstract

**Figure:**
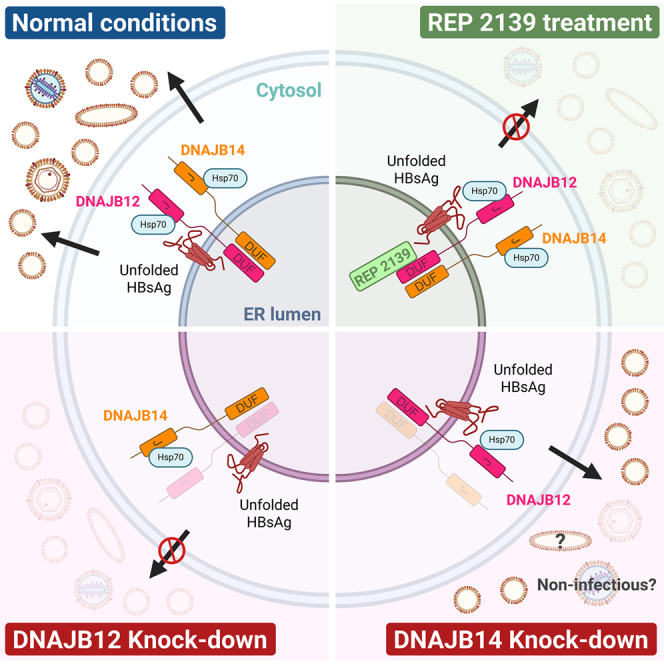

## Introduction

Hepatitis B virus (HBV) chronically infects over 254 million people globally, remaining a significant health challenge. Chronic HBV infection increases individual risks for liver fibrosis, cirrhosis, and hepatocellular carcinoma (HCC), leading to approximately 1 million deaths annually.^1^ During infection, the most predominant circulating antigen is the hepatitis B surface antigen (HBsAg), which corresponds to the viral envelope proteins. HBsAg assembles into non-infectious subviral particles (SVPs), which are empty envelopes produced and secreted in large excess compared to infectious HBV virions, known as Dane particles.^2^ Upon infection, HBV cccDNA persists in the nucleus as a non-integrated mini chromosome-like DNA.^3^ While eradicating cccDNA to achieve a sterilizing cure is challenging, the current goal is to achieve a functional cure,^4^^,^^5^ defined by undetectable circulating HBV DNA and HBsAg after a finite treatment course.^6^^,^^7^ Current treatments for chronic hepatitis B rely on third-generation nucleos(t)ide analogs (NUCs), which target the viral polymerase. While they have demonstrated an effective reduction in serum HBV DNA,^5^ minor to no effect is observed on secreted HBsAg.^8^ Notably, several studies have revealed that HBsAg seroclearance is associated with favorable long-term clinical outcomes.^9^^,^^10^^,^^11^ However, even with prolonged treatment, HBsAg clearance is rare (0–5%),^8^^,^^12^ and frequent viral rebound occurs upon therapy withdrawal, necessitating lifelong treatment.^13^

Hepatitis delta virus (HDV) is a defective virus that requires HBV envelope proteins to form mature infectious virions.^14^ Chronic carriers of HBV and HDV (12–72 million) are at increased risk of developing cirrhosis and HCC.^15^^,^^16^ Currently, treatments for patients with HDV are limited, relying mostly on pegylated interferon alfa-2a, as NUCs do not affect HDV infection, and their efficacy is suboptimal.^17^^,^^18^

Given these challenges, it is crucial to gain insight into HBsAg synthesis and secretion to develop effective treatments and achieve a functional cure for both HBV and HBV/HDV carriers.

Nucleic acid polymers (NAPs), amphipathic single-stranded phosphorothioated oligonucleotides, have shown promising results in phase IIA clinical trials for treating HBV mono-infected and HBV/HDV co-infected patients. The clinically active lead compound, REP 2139, has demonstrated a rapid clearance of HBsAg from circulation. When combined with immunotherapies such as pegylated interferons and NUCs such as tenofovir disoproxil fumarate, REP 2139 achieved functional control of HBV and HDV, resulting in a functional cure for many patients.^19^^,^^20^^,^^21^ Although the mechanism of action of NAPs has been investigated,^22^^,^^23^ it remains unclear.

HBV envelope proteins are synthesized at the endoplasmic reticulum (ER) membrane. HBsAg is a membrane-spanning glycoprotein with three isoforms: small (S-HBsAg), medium (M-HBsAg), and large (L-HBsAg). All these proteins contain the S domain and differ by the presence of additional N-terminal sequences. S-HBsAg consists of the S domain only, while M-HBsAg contains an additional preS2 domain, and L-HBsAg contains an additional preS1 sequence.^24^ All three HBsAg isoforms co-translationally integrate into the ER membrane and contain several transmembrane domains.^25^ Both Dane particles and SVPs carry identical HBsAg, though their protein composition differs, with virions having much higher amounts of L-HBsAg.^26^ L-HBsAg has a multifunctional role, notably involved in virion attachment/entry to hepatocytes and capsid envelopment, through a dual topology. Its N-terminal domain can be post-transcriptionally translocated to either the cytosol (i-preS) or the ER lumen (e-preS).^27^^,^^28^ While HBsAg structure is well characterized (for review see^29^), the host factors and chaperones involved in the maturation and egress processes are essentially reported post-HBsAg assembly and release from the ER (for review see^30^). Chaperones involved in HBsAg folding at the ER remain largely unknown.

Molecular chaperones play a crucial role in folding nascent proteins, preventing protein aggregation, participating in protein quality control, and directing misfolded proteins toward degradation pathways.^31^ The heat shock proteins 70 (Hsp70) family is a major ubiquitous class of chaperones involved in these biological processes.^31^ They require key cofactors to function: Hsp40 proteins (also known as J-proteins or DNAJ proteins).^31^^,^^32^ Hsp40 proteins have a conserved J-domain that interacts with the Hsc/Hsp70 ATPase domain and regulates ATPase activity.^33^^,^^34^ They interact with a non-native polypeptide (also called “client”) and transfer it to the polypeptide-binding site of Hsc/Hsp70.^35^

In this study, REP 2139 was used as a bait to identify cellular targets affecting HBsAg. The analysis of the MS/MS results identified several potential NAP targets, including DNAJB12, a novel ER transmembrane protein part of the Hsp40 family that acts as a co-chaperone with Hsc/Hsp70 and forms heterodimers with DNAJB14, a highly similar protein. Although DNAJB12 and DNAJB14 are still poorly studied, they are known to have their J-domain facing the cytosol.^36^ DNAJB12 is reported to be involved in the proteasomal degradation of misfolded cystic fibrosis transmembrane conductance regulator (CFTR).^37^^,^^38^^,^^39^ DNAJB12/DNAJB14 complexes are reported to promote the membrane penetration of viruses,^40^^,^^41^ facilitate tetrameric assembly of K^+^ ion channels,^42^ and regulate HTT-polyQ aggregations.^43^ We hypothesized that HBsAg may be a client of DNAJB12.

Here, we report a critical role of these two co-chaperones in both HBV and HDV particle morphogenesis and identify DNAJB12 as a REP 2139 target. Our results revealed that DNAJB12 strongly interacts with REP 2139 and, when knocked down (KD), leads to a drastic decrease (>90%) in the morphogenesis of SVPs, Dane particles, and HDV virions, consistent with REP 2139 antiviral effects. Interestingly, DNAJB14 does not interact with REP 2139 and has a lesser effect on SVP morphogenesis, while still exerting an effect comparable to DNAJB12’s on HBV and HDV virions.

## Results

### REP 2139 interactome reveals DNAJB12

**Figure 1:**
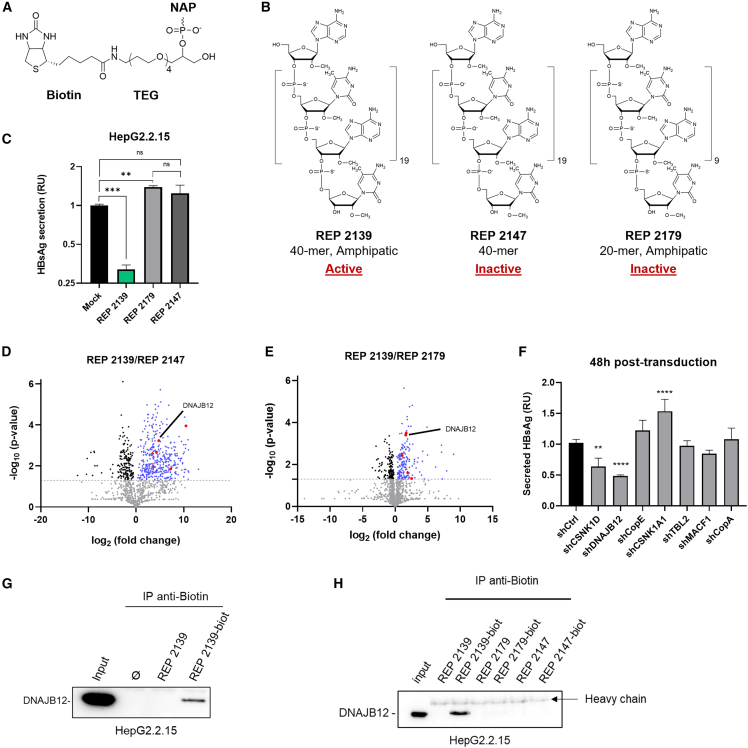
Interactome of REP 2139 and identification of DNAJB12 (A) Structure of the Biotin-TEG ligand. (B) Biochemical structures and features of NAPs used in this study. HepG2.2.15 lysates were incubated with REP 2139 and negative control NAPs, REP 2147, and REP 2179. (C–E) Comparative antiviral effect of REP 2139 and the negative controls REP 2179 and REP 2147 on HBsAg secretion (*n* = 3) (one-way ANOVA, followed by Tuckey’s comparison test); (D) enrichment of proteins interacting with REP 2139 compared to REP 2147 (*n* = 3); and (E) enrichment of proteins interacting with REP 2139 compared to REP 2179 (*n* = 3). (F) Secreted HBsAg at 48 h post-transduction of shRNA targeting various REP 2139-interacting proteins (*n* = 3). Relative to shCtrl (one-way ANOVA, followed by Dunnett’s comparison test). (G) Pull-down of REP 2139-biot followed by WB anti-DNAJB12 in HepG2.2.15 lysates (*n* = 5). (H) Pull-down of REP 2139-biot, REP 2179-biot, and REP 2147-biot followed by WB anti-DNAJB12 in HepG2.2.15. Ø: cell lysate and beads. ∗*p* = 0.05; ∗∗*p* = 0.01; ∗∗∗*p* = 0.001; ∗∗∗∗*p* = 0.0001; ns: non-significance. RU, relative units. Data are represented as mean ± SD. See also Table S1 and Figure S1.

To identify potential REP 2139 targets involved in HBsAg secretion or assembly, we used biotinylated NAPs as baits in pull-down experiments. The structure of the Biotin-TEG (triethylene glycol) spacer ligand used for labeling NAPs is depicted in Figure 1A. NAPs’ antiviral activity relies on their length (optimally ∼40-mer) and their amphipathic nature given by phosphorothioation.^22^ Two inactive NAPs served as negative controls: REP 2147 and REP 2179 (Figures 1B and 1C). Biotinylated NAPs were incubated with HepG2.2.15 lysates, and NAP interactomes were purified using magnetic streptavidin beads and analyzed by Nano LC-MS/MS. Results are presented as enrichment ratios of proteins found in the REP 2139 fraction compared to the controls REP 2147 (Figure 1D) and REP 2179 (Figure 1E). Several proteins were selected based on cellular localization and functions compatible with an involvement in HBsAg secretion/assembly (Table S1). Notably, no viral proteins were found to interact with NAPs. To assess the impact of these proteins on HBsAg secretion, shRNAs were used to KD the identified targets (Figure S1). A primary screening on the best putative targets was performed at 48 h post-transduction of shRNAs (Figure 1F). CSNK1D and DNAJB12 KD resulted in a decrease in HBsAg secretion, with a more pronounced effect observed with DNAJB12 KD. Since we were screening for a potential target of REP 2139, which itself induces a profound decrease in HBsAg secretion, DNAJB12 was selected for further characterization. The interaction of DNAJB12 with biotinylated-REP 2139 (REP 2139-biot) was also demonstrated by anti-biotin immunoprecipitation (IP) and Western blot (WB), using cell lysate devoid of NAPs and cell lysate incubated with non-biotinylated REP 2139 as negative controls (Figure 1G). An anti-biotin IP with biotinylated and non-biotinylated inactive NAPs 2147 and 2179 was also performed to confirm the specificity of the interaction of DNAJB12 with REP 2139-biot (Figure 1H).

### DNAJB12 is essential for HBsAg and subviral particles morphogenesis

**Figure 2:**
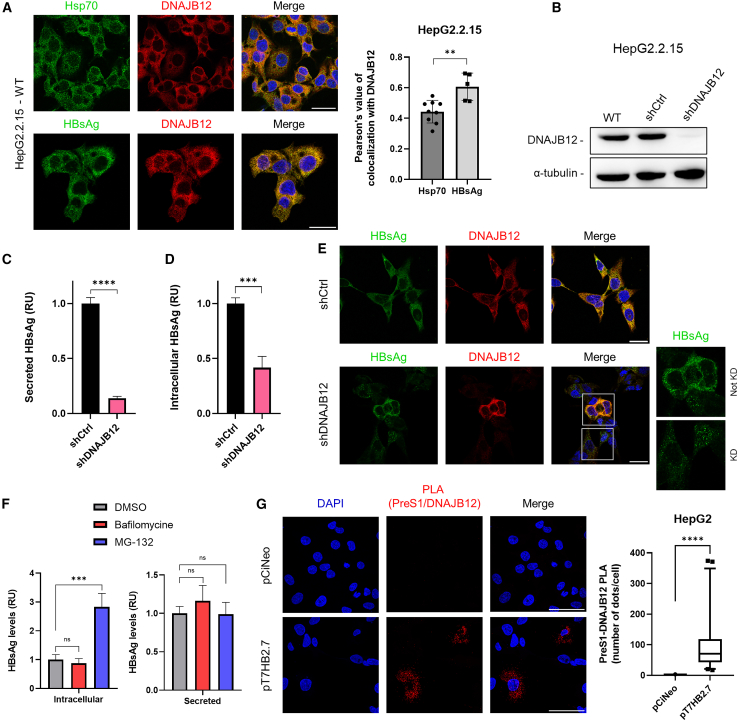
DNAJB12 is essential for HBsAg formation and SVP secretion (A) Colocalization of Hsp70, HBsAg, and DNAJB12 in HepG2.2.15. Scale bars, 25 μm (*n* = 8). (Pearson’s correlation, unpaired t-test, two-tailed). (B–D) WB analysis of HepG2.2.15 depleted of DNAJB12 (shDNAJB12) or control cells (shCtrl) at 5 days post-transduction of shRNA (*n* = 10). Secreted HBsAg (C) and intracellular HBsAg (D) from HepG2.2.15 depleted of DNAJB12 (shDNAJB12) or control cells (shCtrl) at 5 days post-transduction of shRNA (*n* = 3) (unpaired t-test, two-tailed). Relative to shCtrl. (E) DNAJB12 KD HepG2.2.15 (shDNAJB12) or control cells (shCtrl) immunostained for HBsAg and DNAJB12 (*n* = 3). Scale bars, 25 μm. (F) HepG2.2.15 treated with DMSO (negative control), Bafilomycin, or MG-132 and intracellular HBsAg and secreted HBsAg levels quantification (*n* = 3). Relative to DMSO (one-way ANOVA followed by Dunnett’s multiple comparison test). (G) PLA between DNAJB12/PreS1 in HepG2 cells transfected with pCiNeo or pT7HB2.7 (100 cells/condition from 2 distinct experiments). Scale bars, 50 μm. (Mann-Whitney). ∗*p* = 0.05; ∗∗*p* = 0.01; ∗∗∗*p* = 0.001; ∗∗∗∗*p* = 0.0001; ns: non-significance. RU, relative units. Data are represented as mean ± SD. See also Figure S2.

We then investigated the functional link between DNAJB12 and HBsAg. Because DNAJB12 acts as a co-chaperone with Hsp/Hsc70,^31^^,^^32^ the colocalization of DNAJB12 with HBsAg was evaluated in HepG2.2.15, using the colocalization of DNAJB12 with Hsp70 as a comparative. A great colocalization was observed between DNAJB12 and Hsp70. A strong colocalization signal was also observed for DNAJB12 and HBsAg (Figure 2A). To further characterize the impact of DNAJB12 KD on HBsAg secretion, cells were transduced with shRNA-expressing lentiviral vectors. Two other shRNA targeting DNJAB12 were tested, and the shDNAJB12 with the greatest effect on DNAJB12 levels was selected for further analysis (Figure S2). At day 5 post-transduction of shDNAJB12, cell lysates were analyzed by WB, and confirming the KD of DNAJB12 (Figure 2B), accompanied by a drastic decrease in HBsAg secretion as observed by ELISA (Figure 2C). Interestingly, this was not associated with the intracellular accumulation of HBsAg (Figure 2D), suggesting a morphogenesis role of DNAJB12 in addition to the involvement in the secretion of HBsAg. To confirm that the HBsAg intracellular decrease is related to DNAJB12 KD, immunofluorescence was performed on KD and control cells. Cells properly depleted of DNAJB12 display a decrease in HBsAg, while cells that were not depleted of DNAJB12 do not show any HBsAg decrease (Figure 2E). To identify degradation pathways involved in HBsAg degradation, the proteasome inhibitor MG-132 and the lysosome inhibitor bafilomycin A were used. Results demonstrate that no significant difference was observed at the extracellular HBsAg levels, which can be due to the much higher levels of secreted HBsAg (∼50-fold superior to intracellular HBsAg levels), making small secretion variations insignificant. However, while Bafilomycin A did not affect intracellular HBsAg levels, a 3-fold intracellular accumulation of HBsAg was observed upon proteasome inhibition (Figure 2F), consistent with previously identified DNAJB12’s functions within the ERAD degradation pathway.^37^^,^^38^^,^^39^ Moreover, interaction between DNAJB12 and the PreS1 domain of L-HBsAg was confirmed by proximity ligation assay (PLA) in HepG2 cells either transfected with plasmid pT7HB2.7 (expressing L-, M, S-HBsAg) or pCiNeo (negative control) (Figure 2G). Altogether, these results suggest a crucial role for DNAJB12 in the maturation of HBsAg.

### DNAJB14 is not essential for subviral particles morphogenesis

**Figure 3:**
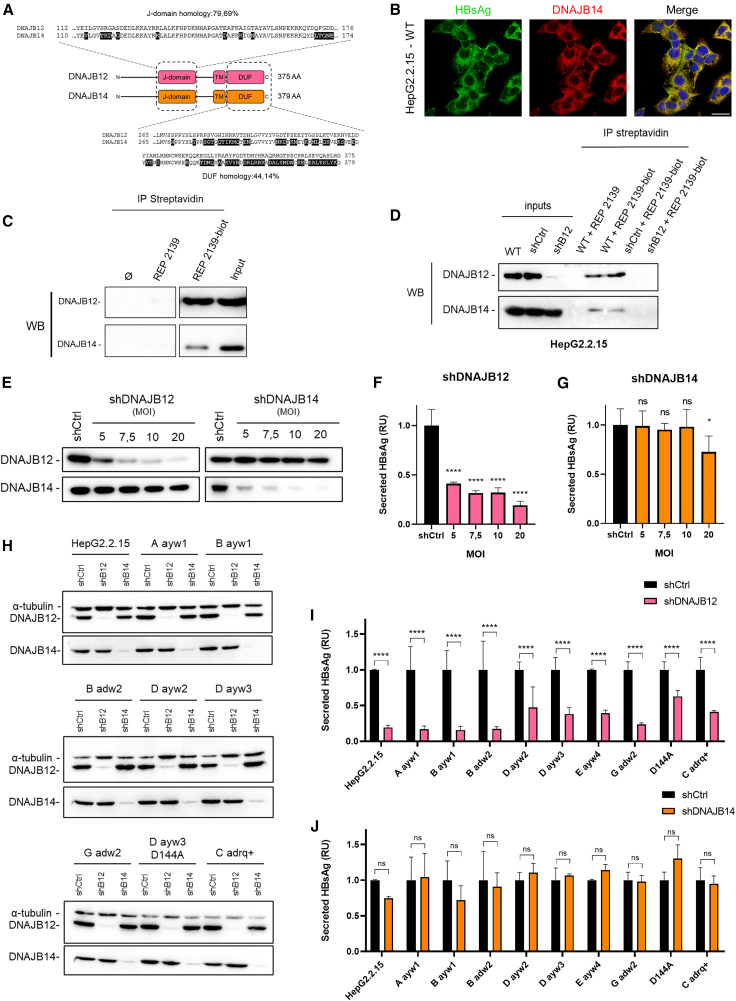
DNAJB14 is not essential for SVP secretion, and DNAJB12 and DNAJB14's effects on HBsAg are pangenotypic (A) Sequence comparison between DNAJB12 and DNAJB14’s J-domain and DUF. (B) Colocalization of HBsAg and DNAJB14 in HepG2.2.15. Scale bars, 25 μm (*n* = 3). (C) Pull-down of biotinylated-REP 2139 (REP 2139-biot) or the non-biotinylated REP 2139 (negative control) followed by WB in HepG2.2.15 lysates (*n* = 3). Ø: cell lysate and beads. (D) HepG2.2.15 KD for DNAJB12 (shB12), or KD control (shCtrl) were lysed and incubated with REP 2139-biot or REP 2139 (negative control) and subjected to a pull-down, followed by WB. (E–G) WB analysis of HepG2.2.15 at 5 days post-transduction of lentiviral particle producing shRNA against DNAJB12 (shDNAJB12), DNAJB14 (shDNAJB14), or unrelated sequence (shCtrl) with various MOI. Secretion of HBsAg from cells in D KD with shDNAJB12 (F) and shDNAJB14 (G) (*n* = 6). Relative to shCtrl. (H–J) Cell lines expressing various genotypes of L-, M-, and S-HBsAg KD for DNAJB12 (shB12), DNAJB14 (shB14) or control cells (shCtrl), cell lysates analyzed by WB. Secreted HBsAg from genotype cell lines in A, KD for DNAJB12 (I) or DNAJB14 (J) (*n* = 3). Relative to shCtrl of each cell line (two-way ANOVA followed by Tukey’s multiple comparison test). ∗*p* = 0.05; ∗∗*p* = 0.01; ∗∗∗*p* = 0.001; ∗∗∗∗*p* = 0.0001; ns: non-significance. RU, relative units. Data are represented as mean ± SD. See also Figure S3.

Several studies have highlighted that DNAJB12 can either work as homodimers^42^ or as heterodimers with DNAJB14,^40^^,^^43^ a highly similar protein to DNAJB12.^36^ While DNAJB12 appears to have a crucial role in the morphogenesis of HBsAg, we wondered about the involvement of DNAJB14 in HBsAg morphogenesis. With an overall identity of 50% between the two co-chaperones, the identity is higher in the J-domain (80%), while lower for the intraluminal “domain of unknown function” (DUF) (44%) (Figure 3A). Structural comparison of DNAJB12 and DNAJB14’s DUF is presented (Figure S4). DNAJB14/HBsAg colocalization was confirmed by immunofluorescence (Figure 3B). While DNAJB14 was not found in the MS/MS results, pull-down experiments with REP 2139-biot followed by WB were performed, and DNAJB14 was detected to a lower extent alongside DNAJB12 (Figure 3C). Two shRNA targeting DNJAB14 were tested, and the shDNAJB14 with the greatest effect on DNAJB14 levels was selected for further analysis (Figure S3). Because DNAJB12 and DNAJB14 can interact as heterodimers,^40^^,^^43^ we wondered if REP 2139 was interacting with both DNAJB12 and DNAJB14 or only with DNAJB12, dragging DNAJB14 along as part of a heterodimer. Pull-down experiments were performed on HepG2.2.15 lysates depleted of DNAJB12. WB results show an absence of DNAJB14 when DNAJB12 is depleted, suggesting an indirect interaction of REP 2139-biot with DNAJB14 via DNAJB12 (Figure 3D). Due to their high homology, we first confirmed by WB that shRNA targeting DNAJB12 was not affecting DNAJB14 and vice versa (Figure 3E). DNAJB14’s effect on HBsAg secretion was assessed in the corresponding supernatants by ELISA. HBsAg secretion was significantly impaired by DNAJB12 KD at every MOI tested (Figure 3F), while DNAJB14 only exhibited a slight effect at the highest MOI (Figure 3G).

### DNAJB12 and DNAJB14’s effects on HBsAg are pangenotypic

To confirm the effect observed on HBsAg by DNAJB12 or DNAJB14 KD, we analyzed their involvement in HBsAg secretion from various HBV genotypes. For that purpose, previously published genotype cell lines (GCLs), expressing the L-/M-/S-HBsAg of 8 different genotypes/serotypes plus a vaccine escape mutant,^44^ were used. The efficiency of DNAJB12 and DNAJB14 KD in the GCL was confirmed by WB (Figure 3H). Corresponding supernatants were analyzed by ELISA for HBsAg secretion. DNAJB12 KD induced a significant decrease in HBsAg secretion for every genotype tested (Figure 3I), while DNAJB14 KD had no significant effect on any of the genotypes tested (Figure 3J). Interestingly, this pangenotypic effect of DNAJB12 on HBsAg secretion is consistent with the previously published REP 2139 pangenotypic effect.^44^ To further assess the pangenotypic involvement of DNAJB12 on SVP secretion, an alignment of L-HBsAg isoforms from each genotype was performed (Figure S4). The alignment highlights highly conserved domains among genotypes, especially transmembrane (TM) domains, which could indicate HBsAg/DNAJB12-interacting regions. Altogether, these results suggest that DNAJB14 is not important for HBsAg formation and SVP morphogenesis, regardless of the genotype, while DNAJB12 appears to be important for every genotype.

### REP 2139 prevents DNAJB12 interactions with HBsAg

**Figure 4:**
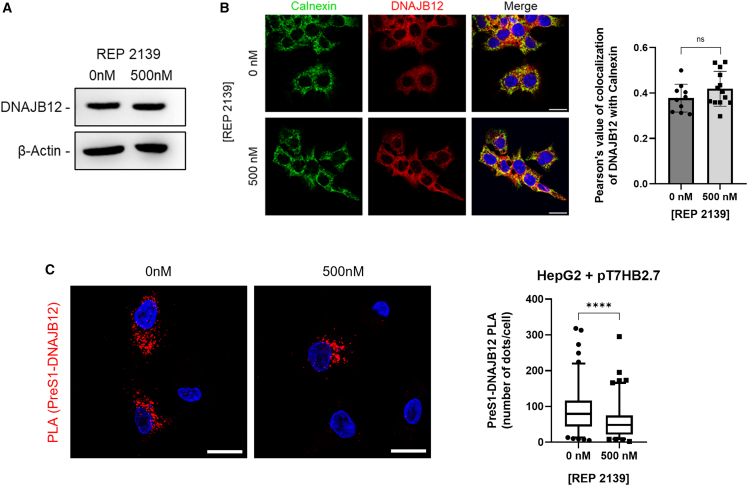
REP 2139 interferes with DNAJB12’s functions (A) HepG2.2.15 treated with 0 nM or 500 nM of REP 2139. DNAJB12 levels in cell lysates were assessed by WB (*n* = 3). (B) Colocalization of DNAJB12 and calnexin in HepG2.2.15 (*n* = 3) (Pearson’s coefficients, unpaired t-test, two-tailed). (C) PLA between DNAJB12/PreS1 in HepG2 transfected with pT7HB2.7 ± 500 nM REP 2139 (Mann-Whitney) (100 cells/condition from 2 distinct experiments). Scale bars, 25 μm ∗∗∗∗*p* = 0.0001; ns: non-significance. RU, relative units. Data are represented as mean ± SD.

Although REP 2139 seems to act only through DNAJB12, the mechanism of action is still unclear. To assess the effect of REP 2139 on DNAJB12 levels, WB experiments were performed on HepG2.2.15 treated with 0 or 500 nM of REP 2139. Results demonstrate that REP 2139 does not affect DNAJB12 levels (Figure 4A), suggesting that NAP's mechanism is not through DNAJB12’s degradation. We then thought to assess the cellular localization of DNAJB12, predominantly ER-localized, after NAP treatment. Results did not show any evidence of DNAJB12 relocalization due to NAP treatment, as confirmed by Pearson’s coefficients of DNAJB12 with calnexin, an ER marker (Figure 4B). Because REP 2139 did not seem to affect DNAJB12’s cellular levels nor its localization, we hypothesized that REP 2139 could act by preventing DNAJB12’s chaperone functions. DNAJB12/L-HBsAg contacts were assessed by PLA, and results demonstrate that NAP treatment decreased the number of interactions between DNAJB12 and L-HBsAg by 32%. This result can only be underestimated, as our analysis does not include cells where all interactions were prevented by REP 2139, as we counted only cells where red dots were visible (Figure 4C). This suggests that REP 2139 interferes with DNAJB12 and prevents it from interacting with HBsAg.

### REP 2139 interacts with DNAJB12 through its luminal domain

**Figure 5:**
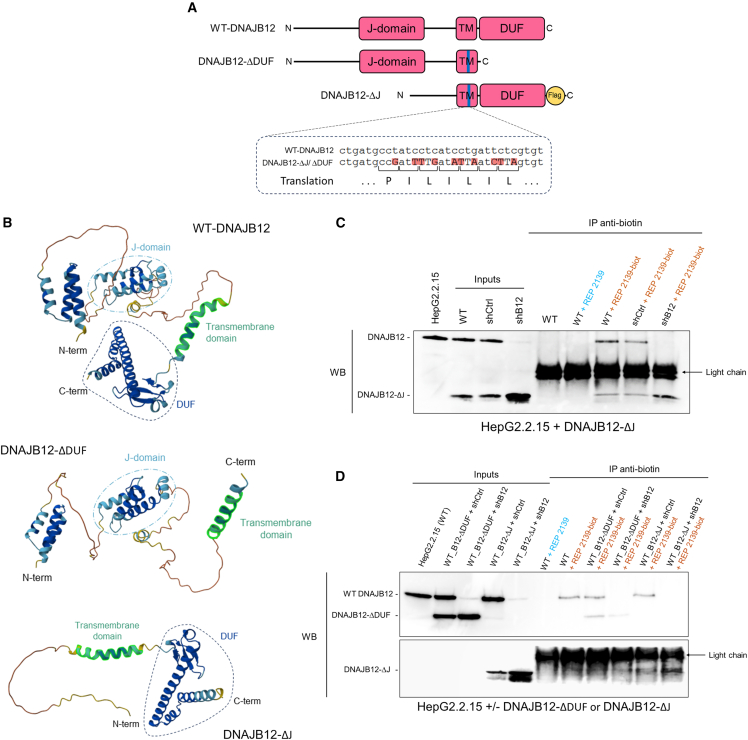
REP 2139 interacts with DNAJB12 via DNAJB12’s intraluminal domain (A) Nucleic acid sequence comparison between endogenous WT-DNAJB12 and sh-resistant truncated isoforms of DNAJB12, showing a silent mutation in translation. (B) 3D-structures of WT-DNAJB12 and truncated DNAJB12 generated by Alphafold. (C and D) HepG2.2.15 expressing the DNAJB12-ΔJ or DNAJB12-ΔDUF isoforms were KD for DNAJB12 (shB12), or KD control cells (shCtrl), and cell lysates were incubated with REP 2139-biot or the REP 2139 and subjected to a pull-down, followed by WB (*n* = 3; *n* = 2).

To better understand REP 2139 interaction with DNAJB12, we aimed to identify the localization of the interaction, whether it is cytosolic or ER-intraluminal. A naturally occurring short isoform of DNAJB12 (ENST00000461919),^43^ lacking the major part of the cytosolic domain and notably the J-domain (named DNAJB12-ΔJ), was used to create a stable cell line via lentiviral transduction. Similarly, another cell line was created with a truncated version missing the DUF (amino acids 1–269 of WT DNAJB12) and named DNAJB12-ΔDUF. Sequences were modified to include silent mutations to resist KD by shDNAJB12 (Figure 5A). Because of their amphipathic nature, it is believed that NAPs interact with α-helix.^45^ 3D representations of the WT endogenous version of DNAJB12 and the truncated isoforms were generated with Alphafold (Figure 5B). The 3D structures show the presence of α-helix in the cytosolic part (especially in the J-domain), as well as in the DUFs. Moreover, modifications to the sequence of the short isoforms did not seem to alter the 3D structures. We first assessed if the REP 2139 interaction with DNAJB12 would be intraluminal, using HepG2.2.15+DNAJB12-ΔJ depleted with WT endogenous DNAJB12. Cell lysates were incubated with REP 2139-biot and non-biotinylated REP 2139 as a negative control. Anti-biotin pull-downs were performed, and the pelleted proteins were analyzed by WB (Figure 5C). When endogenous DNAJB12 was depleted, DNAJB12-ΔJ was detected strongly upon REP 2139-biot pull-down, suggesting that the interaction of REP 2139 with DNAJB12 is through the DUF. The same experiment was performed with HepG2.2.15 +/− DNAJB12-ΔDUF to assess the importance of the cytosolic portion of DNAJB12 (Figure 5D). Noteworthy, DNAJB12-ΔDUF can be pulled down by REP 2139. However, in cells depleted of endogenous WT DNAJB12, the intensity of the DNAJB12-ΔDUF protein band gets weaker when compared to the extract containing WT DNAJB12. This strongly suggests that the DNAJB12-ΔDUF pull-down is occurring through protein-protein interaction and not through a direct interaction with REP 2139. Indeed, it was previously reported that DNAJB12 oligomerization can also occur through its G/F-domain located in the cytosol.^42^ As DNAJB12 and DNAJB14’s G/F-domains share high identity,^42^ we propose that DNAJB12-ΔDUF forms oligomers through its G/F-domain with DNAJB12 and DNAJB14, therefore pulling down DNAJB14, and that the main interaction of REP 2139 with DNAJB12 occurs through its intraluminal DUF.

### DNAJB12 and DNAJB14 are essential for the formation of infectious hepatitis B virus and Hepatitis delta virus virions

**Figure 6:**
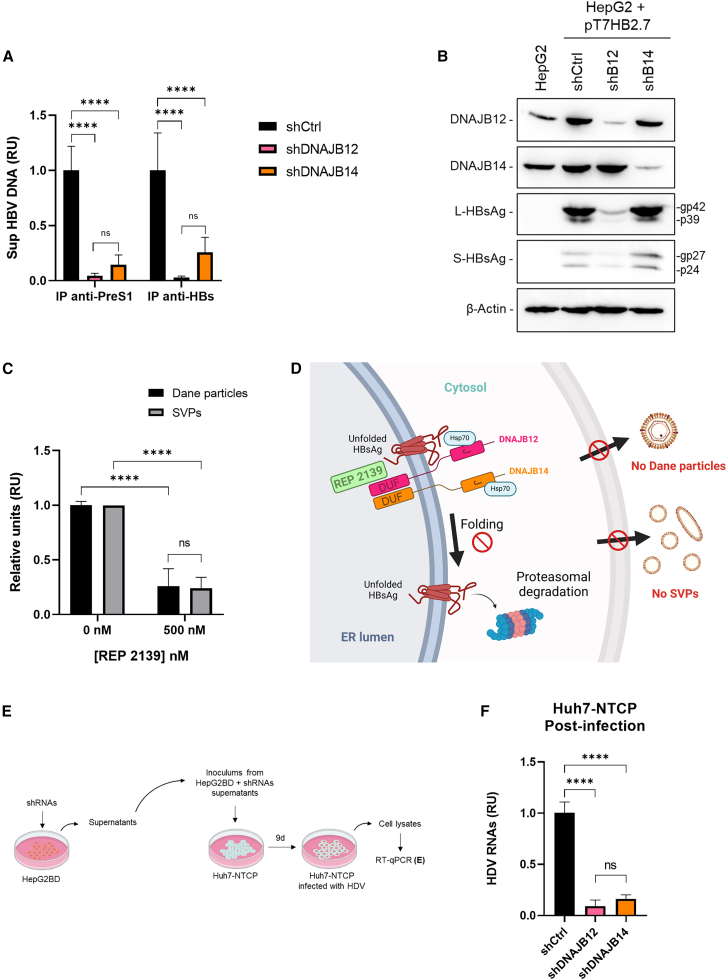
DNAJB12 and DNAJB14 are crucial for the formation of HBV and HDV virions (A) HepG2.2.15 cells were KD for DNAJB12 (shDNAJB12), DNAJB14 (shDNAJB14), or KD control cells (shCtrl), supernatants were collected, and virions were immunoprecipitated with anti-PreS1 or anti-HBs. HBV DNA was assessed by qPCR (*n* = 3) (two-way ANOVA, Tukey’s multiple comparison test). (B) HepG2 cells were KD for DNAJB12 (shB12), DNAJB14 (shB14), or KD control cells (shCtrl) and were transfected with pT7HB2.7 (expressing L-, M-, S-HBsAg). Cell lysates were analyzed by WB (*n* = 3). (C) HepG2.2.15 cells were treated with 0 nM or 500 nM of REP 2139. Supernatants were harvested, treated with benzonase, and Dane particles were IP with anti-PreS1. HBsAg levels were analyzed by ELISA, and HBV DNA was quantified by qPCR (*n* = 4). Results are presented relative to 0 nM of REP 2139 (two-way ANOVA followed by Tukey’s multiple comparison test). (D) Graphical representation of REP 2139 mechanism of action. (E) Experimental design for E is as indicated. (F) Huh-NTCP(L) cells were infected with supernatants from HepG2BD cells KD for DNAJB12 (shDNAJB12), DNAJB14 (shDNAJB14), or KD control cells (shCtrl). At 9 days post-infection, cellular RNAs were harvested and HDV RNA levels were analyzed by RT-qPCR (*n* = 3) (two-way ANOVA followed by Sidak’s multiple comparison test). ∗∗∗∗*p* = 0.0001; ns: non-significance. RU, relative units. Data are represented as mean ± SD.

Because of the crucial effect of DNAJB12 on HBsAg formation, we wondered if it could affect Dane particle morphogenesis. We concomitantly investigated the putative effect of DNAJB14 on Dane particle maturation. As previously mentioned, Dane particles and SVPs carry the same envelope proteins, but their composition differs, with a higher proportion of L-HBsAg in Dane particles.^26^ We first thought to use IP anti-PreS1 to identify Dane particles. However, previous studies have demonstrated that a region of the PreS1-domain of L-HBsAg (i) (cytosolic i-PreS conformation, intra-capsid) is required for the formation of mature HBV virions.^46^^,^^47^ We hypothesized that the KD of DNAJB12 or DNAJB14 could affect the whole formation of the L-HBsAg or its various topologies. To avoid drawing misleading conclusions, IP anti-HBs were conducted along with IP anti-PreS1 to detect all potential virion forms regardless of L-HBsAg topology. HepG2.2.15 were transduced with shCtrl, shDNAJB12, or shDNAJB14. Immunoprecipitation of Dane particles was performed on supernatants. HBV DNA was detected by qPCR. Interestingly, results demonstrate a drastic decrease in HBV virions when both DNAJB12 and DNAJB14 are depleted (Figure 6A). No difference was observed between the two IPs, suggesting that the dual preS topology of L-HBsAg is not affected by the KDs. To determine if L-HBsAg is quantitatively affected by DNAJB12 or DNAJB14 KD, HepG2 cells were transduced with shRNA and transfected 2 days later with the pT7HB2.7 plasmid. Cells were lysed, and the presence of L-HBsAg and S-HBsAg was assessed by WB (Figure 6B). As expected, DNAJB12 KD affected both S- and L-HBsAg, consistent with previous results of DNAJB12 KD affecting both SVPs and Dane particles. In contrast, DNAJB14 KD did not affect S- nor L-HBsAg levels, nor the glycosylation, as confirmed by the presence of gp27 and gp42, suggesting that the reduced virions’ secretion upon DNAJB14 KD is not due to a depletion of L-HBsAg.

Considering these new results, we reevaluated a previously published result about REP 2139 only affecting SVP secretion.^23^ REP 2139 preventing DNAJB12 functions should also affect Dane particle formation/secretion. Supernatants from HepG2.2.15 treated with 500 nM of REP 2139 were collected, treated with benzonase to eliminate any possible remaining DNA from lysed cells, and subjected to anti-PreS1 IP prior to HBV DNA quantification by qPCR. These new results obtained with benzonase treatment clearly reveal that REP 2139 has a similar effect on both SVPs and Dane particle secretion (Figure 6C). Considering these recent results, we hypothesize that REP 2139 acts by preventing DNAJB12 function on HBsAg folding, leading to the proteasomal degradation of unfolded HBsAg and inhibition of the formation/secretion of both SVPs and Dane particles (Figure 6D).

**Figure 7:**
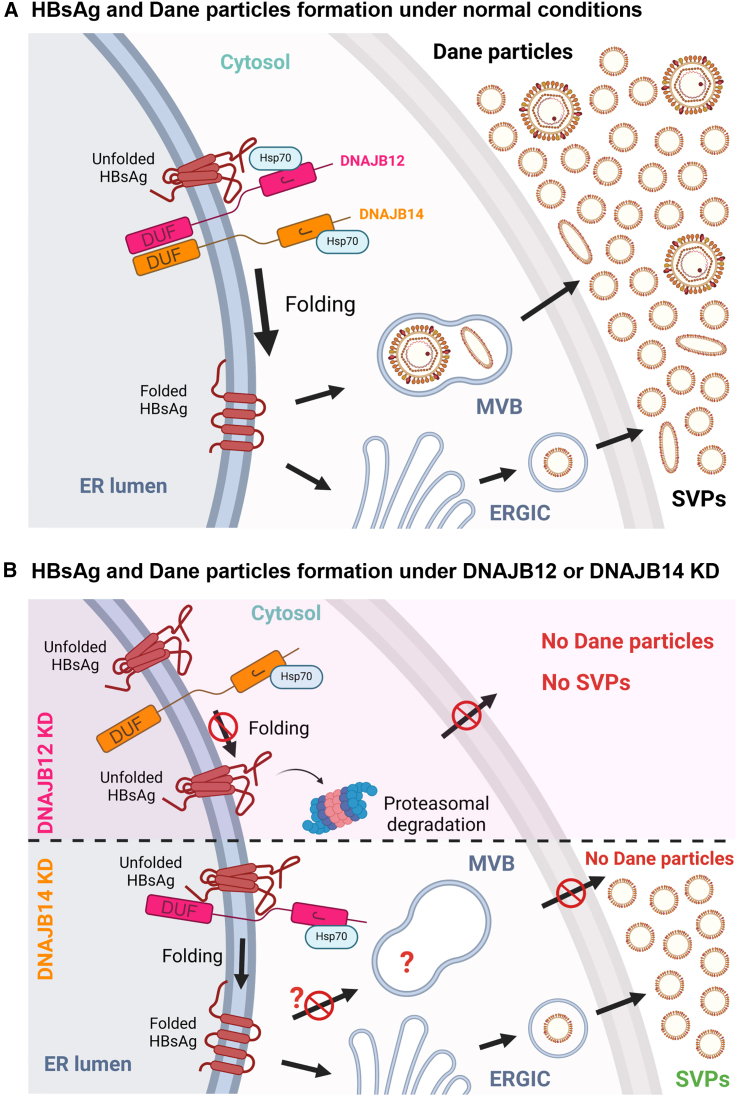
Model for HBsAg folding and secretion pathways (A) HBsAg and Dane particle formation under normal conditions. When DNAJB12 and DNAJB14 are present, L- and S-HBsAg isoforms are properly folded (here, only L-HBsAg is represented). After folding, HBsAg assembles to form either circular SVPs secreted through the ERGIC pathway or filamentous SVPs and Dane particles secreted through the MVB pathway. (B) HBsAg and Dane particles formation under DNAJB12 or DNAJB14 KD. When DNAJB12 is KD, both L- and S-HBsAg isoforms are misfolded and sent to proteasomal degradation, leading to no SVP formation and secretion and no Dane particle or infectious HDV virion (not represented here) formation and secretion. When DNAJB14 is KD, HBsAg isoforms are still properly folded and assemble to form SVPs secreted through the ERGIC pathway. Conversely, no Dane particle nor infectious HDV virions (not represented here) were observed.

HBsAg being needed for the formation of mature HDV virions,^14^ we aimed to determine if the results obtained for HBV virions were transposable to HDV virions. HepG2BD, a novel HepG2.2.15-derived cell line expressing both HBV and HDV,^48^ was transduced with shCtrl, shDNAJB12, or shDNAJB14, and supernatants were collected and used to infect Huh7-NTCP cells. After 9 days, cells were lysed, and HDV RNA levels were quantified by RT-qPCR (Figure 6E). Results display a significant decrease in HDV RNA levels in cells infected with supernatants from DNAJB12 or DNAJB14 KD cells (Figure 6F). A model for normal HBsAg folding, SVP, and Dane particle formation and secretion is suggested in Figure 7A. An alternative model representing the effects of DNAJB12 or DNAJB14 KD is presented in Figure 7B. Altogether, these results reveal the crucial role of both DNAJB12 and DNAJB14 in the morphogenesis of HBV and HDV virions.

## Discussion

In this study, we identify DNAJB12 and DNAJB14 as two critical chaperones for HBV and HDV particle formation.

It was previously reported from clinical trials that NAPs can inhibit HBsAg secretion and HDV virion secretion.^19^^,^^20^^,^^21^^,^^49^ Here, we focused on DNAJB12 due to its strong inhibitory effect on HBsAg secretion, mimicking NAPs' clinical antiviral effects, although studying the other identified targets would be of interest. Notably, KD of CSNK1A1 resulted in a significant increase in HBsAg secretion, and understanding the underlying mechanism behind this effect shall be performed in a future study.

Our findings establish the critical role of DNAJB12 in the formation and secretion of HBsAg. DNAJB12 KD in HepG2.2.15 was associated with a striking decrease in both intracellular and extracellular HBsAg levels. Previous work highlighted the need for a Hsp40 chaperone to assist in the formation of an L-HBsAg/Hsc70 complex, noting that “L-HBsAg shares common features with the CFTR, which is held and folded by the Hsp40/Hsc70 chaperone pair.”^50^ Subsequent studies revealed DNAJB12’s role in CFTR’s fate.^37^^,^^38^^,^^39^ Thus, we propose that DNAJB12 is involved in HBsAg isoforms' folding. Moreover, DNAJB12 could participate in addressing misfolded HBsAg isoforms to the ERAD pathway and the proteasome, consistent with DNAJB12’s previously identified functions^37^^,^^38^^,^^39^ and our results demonstrating intracellular HBsAg accumulation when proteasome inhibition by MG-132.

Furthermore, our pangenotypic alignment suggests the interaction of DNAJB12 and HBsAg, possibly occurring in one of the TM domains. As TM1 and TM2 are known to be crucial for SVP formation,^51^^,^^52^ and the interaction of DNAJB12 with one of those two TM domains could be required for folding and SVP assembly. The pangenotypic effect of DNAJB12 KD is also consistent with previous results demonstrating a pangenotypic antiviral effect of the REP 2139 *in vitro.*^44^

Despite their sequence and structure similarities, our studies revealed that DNAJB14, unlike DNAJB12, does not appear to be crucial for the formation and secretion of SVPs. Recent studies have highlighted that DNAJB12 and DNAJB14 are non-redundant chaperones,^53^ consistent with our findings. Moreover, DNAJBC14, DNAJC18, and DNAJC30 are known to be other homologues of DNAJB12 and DNAJB14, as they are ER-located transmembrane proteins with their J-domain facing the cytosol. Given that several chaperones from the DNAJ family have been demonstrated to play significant roles in various viral infections,^54^^,^^55^^,^^56^^,^^57^ and that DNAJB12/DNAJB14 complexes are also involved in different viral mechanisms,^40^^,^^41^ we considered the roles of DNAJBC14, DNAJC18, and DNAJC30 in the context of viral infections based on the existing literature. DNAJC30 appears to positively regulate HIV-1 replication by modulating the LTR promoter’s activity.^58^ Interestingly, DNAJC14 is particularly important for *Flaviviridae*, with many studies demonstrating the role of DNAJC14 in viral replication and in the proper folding of the polyprotein, among others.^59^^,^^60^^,^^61^^,^^62^^,^^63^^,^^64^^,^^65^ DNAJC18 is also involved in flavivirus replication,^65^ and has been reported to have non-redundant and complementary functions alongside DNAJB12 and DNAJB14 in facilitating SV40 infection.^40^^,^^66^^,^^67^ Further investigation into the interplay between DNAJC18 and DNAJB12/DNJAB14 during HBV infection and its effect on the formation of HBV and HDV particles would be of interest.

REP 2139’s mechanism of action with DNAJB12 was investigated in this study: truncated DNAJB12 isoforms were used to determine the DNAJB12/REP 2139 interaction domain, and results suggest that REP 2139 interacts with the DUF domain of DNAJB12. This interaction might be responsible for the REP 2139 antiviral effect, thereby preventing DNAJB12 functions and HBsAg proper folding. The C-terminal extension of J-proteins, corresponding to the DUF, is known to be a conserved client-binding domain among yeast, bacteria, and humans.^31^^,^^68^^,^^69^^,^^70^^,^^71^^,^^72^^,^^73^ The distal part of the C-terminal domain also contains a dimerization domain that increases the affinity for clients,^31^^,^^68^ which would be consistent with REP 2139 preventing DNAJB12/HBsAg interaction through the DUF. While we cannot exclude that REP 2139’s antiviral effect occurs through another protein and/or another mechanism, our results suggest that REP 2139 likely inhibits DNAJB12 functions by interacting with its DUF. Further experiments shall be performed to confirm that this interaction is necessary for inhibiting DNAJB12 functions.

Our research demonstrated the critical and essential role of both DNAJB12 and DNAJB14 in the formation and secretion of HBV and HDV virions. In DNAJB12-depleted cells, the absence of S- and L-HBsAg consequently prevented virion production, as well as SVP morphogenesis. Conversely, in DNAJB14-depleted cells, S- and L-HBsAg production proceeded normally, consistent with our prior findings that DNAJB14 does not affect SVP morphogenesis and secretion. The fact that only HBV and HDV virions are affected suggests an effect on L-HBsAg, necessary for the assembly of Dane particles,^27^^,^^28^ and both virions’ infectivity. The budding and egress of these viral particles depend on intraluminal vesicles called multivesicular bodies (MVBs), maturing endosomes, and the ESCRT pathway,^74^^,^^75^^,^^76^ while SVPs do not rely on ESCRT and are released through the constitutive secretion pathway.^77^^,^^78^ ESCRT proteins are required for the transport of membrane proteins by forming vesicles that bud inward within the MVBs.^79^ It was previously reported that the secretion of infectious HBV particles via the ESCRT-MVB pathway is mediated by interaction between L-HBsAg and cellular proteins such as α-taxilin^80^ and γ2-Adaptin.^81^ DNAJB14 could be involved in these interactions. Recent research also highlighted that ERGIC-53, a transmembrane protein involved in protein transport between the ER, ERGIC, and Golgi, was crucial for HBV virion secretion but dispensable for SVPs.^82^ We hypothesize that an interaction between DNAJB14 and ERGIC-53 may be essential for HBV virion trafficking. The involvement of ERGIC-53 in the secretion of the HDV virion has not been elucidated yet. However, HDV virions' trafficking relies on the ER-Golgi pathway.^83^ Therefore, as ERGIC-53 is involved in protein transport between these cellular compartments, we speculate that it could also be needed for the secretion of HDV virions. In the absence of DNAJB14, ERGIC-53 would fail to participate in the secretion of both Dane particles and HDV virions. Furthermore, since the envelope of filamentous SVP is composed of the same ratios of S-, M-, and L-HBsAg proteins as Dane particles’ envelope,^26^ it has been shown that filamentous SVPs are also secreted via the same secretion pathway as Dane particles.^74^ Consequently, the knockdown of DNAJB14 may also affect the secretion of filamentous SVP, which is present in smaller quantities compared to spherical SVP. This could explain the slight effect on HBsAg secretion observed upon KD of DNAJB14 at high MOI levels (Figure 3G, MOI = 20). This will be investigated in future work.

Since HDV RNA is significantly reduced in cells infected with supernatants from DNAJB12 and DNAJB14-depleted cells, we conclude that HBV and HDV maturation and/or infectivity are compromised. DNAJB14 KD might not prevent the production of non-infectious HDV virions, lacking the L-HBsAg. This matter should be assessed in future studies.

Our study is the first to underscore the pivotal roles of DNAJB12 and DNAJB14 chaperones in the morphogenesis of both infectious and non-infectious HBV particles, as well as HDV infectious particles. Additionally, as DNAJB12 KD recapitulated REP 2139 antiviral effects observed in clinical trials, DNAJB12 emerged as the potential primary target of REP 2139. These results contribute to a better understanding of HBV and HDV viral morphogenesis and antiviral mechanisms, aiding progress toward HBV and HDV eradication.

### Limitations of the study

The antiviral effect of REP 2139 was characterized primarily in HepG2.2.15 and HepG2 cell lines. While these are suitable models for studying HBV biology and host-virus interactions, they do not fully replicate the complexity of a complete liver or organism. Therefore, conducting *in vivo* experiments would be valuable for further research, but this approach remains challenging because NAPs are inactive in mice.

## Resource availability

### Lead contact

Further information and requests for reagents or resources should be directed to and will be fulfilled by the lead contact, Patrick Labonté (patrick.labonte@inrs.ca).

### Materials availability

Reagents generated in this study will be made available on request, but we may require completing a materials transfer agreement.

### Data and code availability

• All data reported in this article will be shared by the lead contact upon request.
• This article does not report original code.
• Any additional information required to reanalyze the data reported in this article is available from the lead contact upon request.

## Acknowledgments

This work was funded by an Alliance grant from the 10.13039/501100000038Natural Sciences and Engineering Research Council of Canada (NSERC), QC, Canada. P.L. was the recipient of the NSERC-Alliance grant (558342-2020). L.A. was the recipient of a Doctoral scholarship from the 10.13039/100012618Fondation Armand-Frappier. We want to thank Laurent Chatel-Chaix, Clément Mazeaud and Mathilde Broquière for their scientific advice. We thank Terence N. Bukong for the proofreading of the article. We thank Jessy Tremblay for his technical support with the confocal microscopy.

## Author contributions

L.A. and P.L. conceived and designed the experiments. L.A. and R.B. performed the experiments. L.A., R.B., and P.L. analyzed the data. L.A. wrote the original draft, and L.A., R.B., and P.L. reviewed and edited the article.

## Declaration of interests

The authors declare no competing interests.

## STAR★Methods

### Key resources table

**Table:** 

REAGENT or RESOURCE	SOURCE	IDENTIFIER
**Antibodies**		

Anti-HBsAg	Abcam	Ab9193; RRID: AB_307062
Anti-DNAJB12	Sigma	HPA010642; RRID: AB_1078692
Anti-Hsp70	Abcam	Ab2787; RRID: AB_303300
Anti-DNAJB14	Novus Biological	NBP2-92648; RRID: AB_3462549
Anti-calnexin	Invitrogen	MA3-027; RRID: AB_2069043
Anti-biotin	Jackson ImmunoResearch	200-002-211; RRID: AB_2339006
Anti-DNAJB12	Proteintech	16780-1-AP; RRID: AB_2094404
Anti-β-actin	Sigma	A5441; RRID: AB_476744
Anti-α-tubulin	Sigma	T9026; RRID: AB_477593
Anti-HBs	Santa-cruz	Sc-53299; RRID: AB_629595
Anti-PreS1	Santa-cruz	Sc-57761; RRID: AB_783720
normal mouse IgG1	Santa-cruz	Sc-3877; RRID: AB_737222
normal mouse IgG2a	Santa-cruz	Sc-3878; RRID: AB_737242

**Chemicals, peptides and recombinant proteins**		

2-mercaptoethanol	Bio-Rad	1610710
Dimethyl Sulfoxide (DMSO)	Millipore Sigma	D8418
Polyethylenimine, Linear, MW 25000	Kyfora Bio LLC	23966-100
MG-132	Millipore Sigma	474790-1MG
BafilomycinA1	Millipore Sigma	B1793
UNC7938	Gift from R. L. Juliano^84^	N/A
REP 2139	Replicor^22^^,^^23^	N/A
Biotinylated REP 2139	Replicor	This paper
Biotinylated REP 2147	Replicor	This paper
Biotinylated REP 2179	Replicor	This paper

**Critical commercial assay**		

Duolink® *In Situ* Red Starter Kit Mouse/Rabbit	Sigma Aldrich	DUO92101-1KT
QIAamp DNA Mini Kit	Qiagen	51306
GS HBsAg EIA 3.0	Bio-Rad	32591
Aurum total RNA mini kit	Bio-Rad	7326820
QuikChange II XL Site-Directed Mutagenesis Kit	Agilent Technologies	200522

**Experimental models: cell lines**		

Hepg2-derived genotype cell lines	Angelo et al.^44^	N/A
HepG2.2.15	Gift from C. Sureau	N/A
HepG2	Gift from C. Sureau	N/A
HEK293T	Gift from L. Chatel-Chaix	N/A
HeLa	–	–
Huh7-NTCP(L)	Blanchet et al.^48^	N/A
HepG2.2.15-DNAJB12-ΔDUF	This paper	N/A
HepG2.2.15-DNAJB12-ΔJ	This paper	N/A

**Oligonucleotides**		

shRNA: CSNK1D	Sigma Aldrich	TRCN0000010640
shRNA: DNAJB12 (1)	Sigma Aldrich	TRCN0000022294
shRNA: CopE	Sigma Aldrich	TRCN0000381999
shRNA: CSNK1A1	Sigma Aldrich	TRCN0000006042
shRNA: TBL2	Sigma Aldrich	TRCN0000156411
shRNA: MACF1	Sigma Aldrich	TRCN0000055847
shRNA: CopA	Sigma Aldrich	TRCN0000065271
shRNA: DNAJB14 (1)	Sigma Aldrich	TRCN0000150360
shRNA: DNAJB12 (2)	Sigma Aldrich	TRCN0000022295
shRNA: DNAJB12 (3)	Sigma Aldrich	TRCN0000022297
shRNA: DNAJB14 (2)	Sigma Aldrich	TRCN0000150558
shCtrl: non target	Gift from L. Chatel-Chaix	N/A

**Recombinant DNA**		

Plasmid: pSPAX2	Addgene	12260
Plasmid: pMD2G	Addgene	12259
Plasmid: pRSV-REV	Addgene	12253
Plasmid: pWPI-puro	Gift from L. Chatel-Chaix	N/A

**Software and algorithms**		

ChemDraw	Revvity Signals	N/A
Prism 9	GraphPad	N/A
ImageJ (Fiji)	NIH	N/A
AzureSpot	Azure Biosystems	N/A

### Experimental model and subject details

#### Cells and reagents

HepG2.2.15, HepG2, Hepg2-derived genotype cell lines (GLC),^44^ HepG2BD^48^ and Huh7-NTCP(L)^48^ were maintained in William’s medium E (WME) complemented with 10% fetal bovine serum (FBS), gentamicin and GlutaMAX. HEK293T and HeLa cells were cultured in Dulbecco’s modified Eagle medium complemented with 10% FBS and gentamicin. NAPs were solubilized in normal saline. The UNC7938 compound, a gift from Dr. Rudolph L. Juliano,^84^ was resuspended in DMSO (Sigma-Aldrich). All reagents are details in the supplementary CTAT Table.

### Method details

#### Plasmids and transfection

psPAX2, pMD2.G, and pRSV-REV plasmids were purchased from Addgene. pWPI-puro plasmid was obtained from Laurent Chatel-Chaix.^85^ pT7HB2.7 plasmid was a gift from Camille Sureau.^86^

For the creation of HepG2.2.15 cell lines expressing truncated versions of DNAJB12, the sequence of a naturally occurring isoform lacking the J-domain was used for the creation of the “DNAJB12-ΔJ” cell line (ENST00000461919). The “DNAJB12-ΔDUF” sequence was created by inserting a STOP codon in position 270 (P > STOP) of the WT human sequence of DNAJB12. Both sequences were designed to include sh-resistant silent mutations (see Figure 5) and were synthesized and ordered from BioBasic. The sequences were inserted into a pWPI-puro plasmid to produce lentiviral vectors. Lipofectamine 3000 (ThermoFisher) was used for transfections. Stable HepG2.2.15-DNAJB12-ΔDUF or DNAJB12-ΔJ cell lines were selected with puromycin (Sigma-Aldrich).

#### NAPs immunoprecipitation assays and mass spectrometry

For NAPs interactome identification, 4 × 10^5^ HepG2.2.15 cells were lysed in pierce buffer with protease inhibitors (Roche) for each condition. Cell lysates were supplemented with 5 μg of either biotinylated REP 2139, REP 2147 or REP 2179 and incubated for 3 h at 4°C, followed by addition of Dynabeads M-270 Streptavidin beads (Thermofisher) and overnight incubation at 4°C with rotation. The precipitates were washed three times with washing buffer (25 mM Tris-HCl pH 7.4, 150 mM NaCl, 1 mM EDTA, 0.1% Tween20) complemented with protease inhibitors (Roche), followed by 10 washed with PBS. The immunoprecipitants were subjected to LC MS/MS analysis. The intensities of proteins detected for the REP 2139 condition were compared to those detected for the REP 2147 and REP 2179 conditions. Enrichment ratios are presented in Figure 1.

For western blot immunoprecipitation analysis, HepG2.2.15 cells were lysed in pierce buffer with protease inhibitors (Roche). Cell lysates were incubated with 50 nM of biotinylated-NAPs and Dynabeads M-270 Streptavidin beads or Dynabeads Protein G (Thermofisher) coupled with anti-biotin antibody and incubated with rotation overnight at 4°C. The precipitates were washed three times with washing buffer (25 mM Tris-HCl pH 7.4, 150 mM NaCl, 1 mM EDTA, 0.02% Tween20) complemented with protease inhibitors (Roche). The immunoprecipitants were resuspended in 2X Laemmli buffer and submitted to western blot assays.

#### Knockdown by small-hairpin RNAs (shRNAs)

MISSION® pLKO.1 plasmid containing shRNA sequences were purchased from Sigma. Reference numbers and sequences are listed in the supplementary CTAT Table. Lentiviral vectors were produced by transfection of HEK293T cells with pLKO.1-shRNA plasmids, along with packaging plasmids psPAX2, pMD2.G, and pRSV-REV. The supernatants from these transfected cells were collected at day 2 post-transfection, clarified, and filtered (0.45 μm) and titrated in HeLa cells. Targeted cells were seeded at 1,25 × 10^5^ cells/well in 24wp and were transduced the next day with lentiviruses at a multiplicity of infection (MOI) of 20 unless otherwise indicated in figure legends, for 24 h in the presence of 8 μg/ml Polybrene (Sigma).

#### HBV detection

For detection of HBV DNA from secreted Dane particles, benzonase-treated supernatants were complemented with anti-preS1 antibodies (Santa-cruz) or the isotype counterpart (Santa-cruz) along with Protein A/G PLUS-Agarose beads (Santa-cruz). The mix was incubated for 2h at RT under rotation. Samples were washed with PBS prior to lysis and DNA extraction using the QIAamp DNA Mini Kit (Qiagen), followed by qPCR (see supplemental information).

Total cellular RNA was extracted using the Aurum™ Total RNA Mini Kit (Bio-Rad). Purified RNA was then normalized using a nanodrop. Reverse transcription and qPCR were conducted (see supplemental information).

HBsAg in supernatant was measured by ELISA using the GS HBsAg EIA 3.0 kit (Bio-Rad).

#### HDV infection and detection assay

Huh7-NTCP(L) cells were seeded on collagen-coated plates and were infected with inoculums as previously described.^48^ At day 10 post-inoculation, cells were harvested. HDV RNA was extracted using the Aurum™ Total RNA Mini Kit (Bio-Rad), normalized by nanodrop, followed by RT-qPCR.

#### RT-qPCR and qPCR

Reverse transcription was conducted using the iScript™ Select cDNA Synthesis Kit (Bio-Rad) with random hexamers as primers. Taqman qPCR from HDV reverse transcribed RNA was performed as previously described.^48^ HBV DNA (Dane particles) quantification were conducted using the SsoAdvanced™ Universal SYBR® Green Supermix (Bio-Rad). Primers for HBV were as described.^87^ Results were compared relative to a reference sample using the Δct method.

#### Western blot

The proteins were resolved by SDS-PAGE, transferred to PVDF membranes, blocked for 2 h at RT and incubated with primary antibodies overnight at 4°C. Washed membranes were incubated for 2h at RT with secondary HRP-antibodies. Protein bands were visualized with the Clarity western ECL (Bio-Rad) using a Sapphire Biomolecular Imager (Azure Biosystems).

#### Confocal immunofluorescence microscopy

Cells were cultured on collagen-coated glass coverslips and fixed for 10 min in 4% paraformaldehyde. Cells were permeabilized for 30 min with 0.2% TritonX-100, followed by incubation with blocking solution (3% BSA, 10% FBS) for 1 h at RT. Incubation with primary antibodies (2 h at RT) were followed by incubation with Alexa Fluor® 488/568 AffiniPure IgG 1 h at RT. Cell nuclei were stained with DAPI. Coverslips were mounted on microscope slides using Prolong antifade reagent (ThermoFisher). Cells were analyzed using a confocal microscope (Zeiss LSM 780).

#### Proximity ligation assays (PLA)

HepG2 cells were seeded on collagen-coated plates and were transfected with either pT7HB2.7 or pCiNeo (negative control). At 48h post-transfection, cells were trypsinized and reseeded on collagen-coated glass coverslips and fixed the next day with 4% paraformaldehyde. Cells were permeabilized for 30 min with 0.2% TritonX-100. Proximity ligation assays were performed using the Duolink PLA Kit (Millipore-Sigma) according to the manufacturer’s protocol. Briefly, cells were blocked in a humidity chamber for 1 h at 37°C, then incubated with the primary antibodies (anti-PreS1 and anti-DNAJB12 from Sigma) for 2 h at RT. Cells were washed twice and incubated with PLUS and MINUS PLA probes in a humidity chamber for 1 h at 37°C. After two additional washes, cells were incubated with ligation solution in a humidity chamber for 30 min at 37°C and then with the amplification solution for 100 min at 37°C. After the final washes, the coverslips were incubated with the DAPI for 15 min followed by an additional wash with buffer B. Coverslips were mounted on microscope slides using Prolong antifade reagent (ThermoFisher). Cells were analyzed using a confocal microscope (Zeiss LSM 780). Each detected signal represents an interaction event. Image analysis and PLA dot counting were performed with the Fiji software.

For PLA experiment +/- NAPs, HepG2 cells were seeded on collagen-coated plates and were transfected with pT7HB2.7. After 24 h, 0 nM or 500 nM of REP 2139 was added to the cells, followed by a 24 h incubation. UNC7938 was added for 2 h, cells were washed with PBS and further incubated for 6 h. Cells were then trypsinized and reseeded on collagen-coated glass coverslips and fixed the next day with 4% paraformaldehyde. PLA was performed as previously described. The graph in Figure 5C shows the number of PLA dots per cell from a total of 100 cells from two different experiments.

### Quantification and statistical analysis

As indicated in the legends, the statistical analyses performed were either Unpaired Student’s t-test, Mann-Whitney test, One-way or Two-way ANOVA analyses followed by multiple comparison tests. Data are presented as the mean and standard deviation (SD) of the mean of different biological replicates as indicated in the figure legends. Statistical analyses were performed using Prism-GraphPad. For every figure, significance is as follows: ∗p=0.05; ∗∗p=0.01; ∗∗∗p=0.001; ∗∗∗∗p=0.0001.
