# Supplementary material for: Unveiling DNAJB12 and DNAJB14 as crucial chaperones in hepatitis B and D virus particle morphogenesis
Source: iScience. 2025 Oct 30;29(2):113897. doi: 10.1016/j.isci.2025.113897 (PMC12890720; doi:10.1016/j.isci.2025.113897)

iScience, Volume *29*

Supplemental information

Unveiling DNAJB12 and DNAJB14 as crucial chaperones in hepatitis B and D virus particle morphogenesis

Léna Angelo, Richard Boulon, and Patrick Labonté

Supplementary Figures

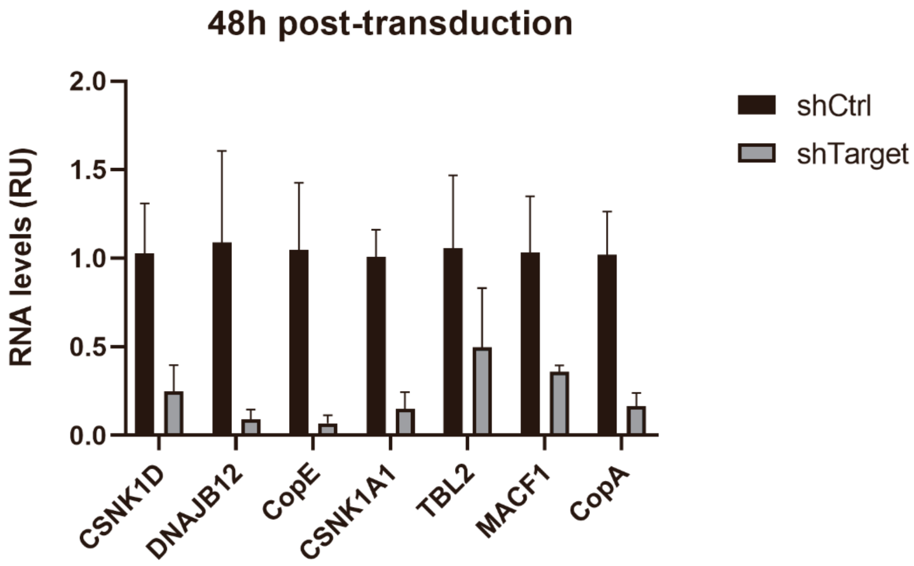

Figure S1. Potential NAPs target proteins RNAs knockdown verification, related to Figure 1.

HepG2.2.15 cells were transduced with shRNA lentiviruses and RNAs levels were assessed 48h post-transduction (n = 3). Results are presented relative to shCtrl. RU, relative units.

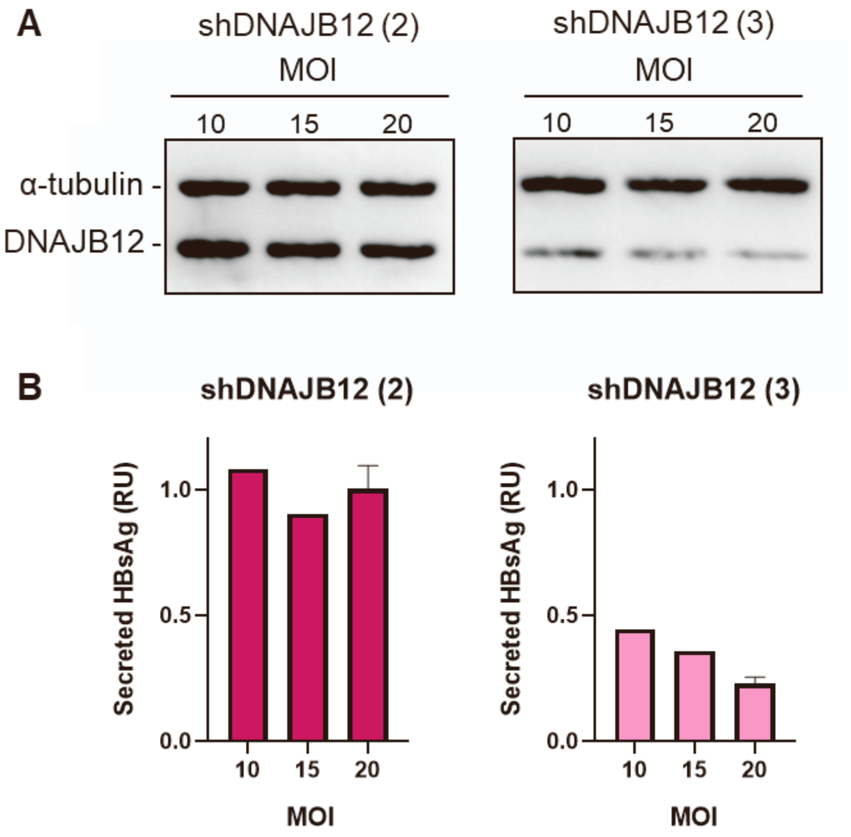

Figure S2. Other shRNA sequences against DNAJB12 tested, related to Figure 2.

(A) HepG2.2.15 cells were transduced with shRNA lentiviruses; cells were lysed 5 days post-transduction and cell lysate were analyzed by western blot. (B) Corresponding HBsAg secretion. RU, relative units. While no effect on DNAJB12 levels is observed in A for sh2 (TRCN0000022295) associated with no decrease in HBsAg secretion levels, results for sh3 (TRCN0000022297) show a dose-dependant decrease of HBsAg levels correlated with DNAJB12 decrease. Since DNAJB12 KD was less efficient with sh2 compared to sh1 (Fig 2 and Fig 3), sh1 (TRCN0000022294) was used in this study.

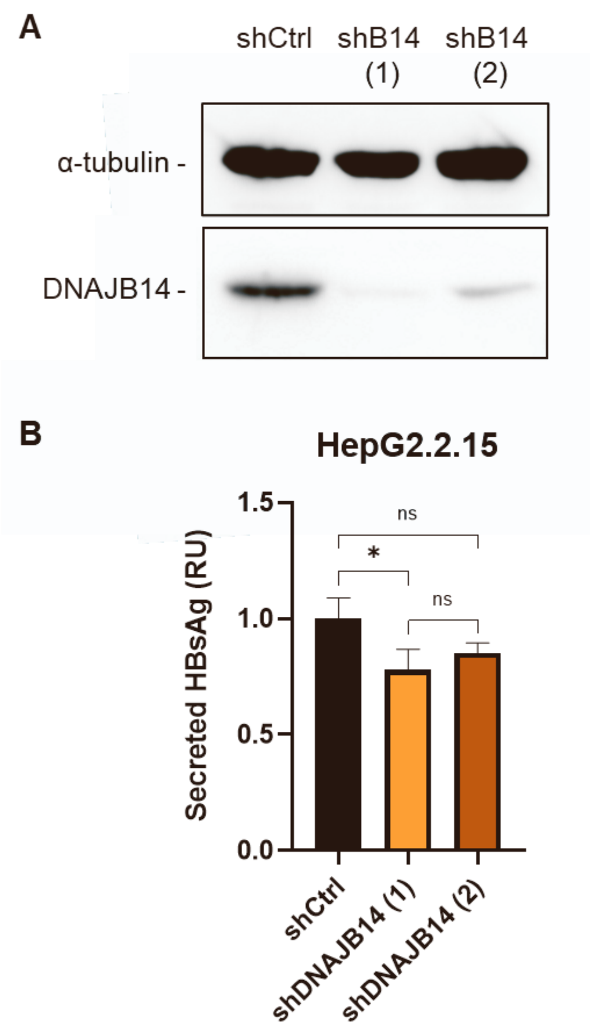

Figure S3. Other shRNA sequence against DNAJB14 tested, related to Figure 3.

(A) HepG2.2.15 cells were transduced with shRNA lentiviruses (MOI = 20), cells were lysed 5 days post-transduction and cell lysate were analyzed by western blot. (B) Corresponding HBsAg secretion. RU, relative units. Both shRNA against DNAJB14 were effective and had similar effect on HBsAg secretion but shB14 (1) (TRCN0000150360) had the greatest effect on DNAJB14 levels and was used in this study (one-way ANOVA followed by Tukey's multiple comparison test).

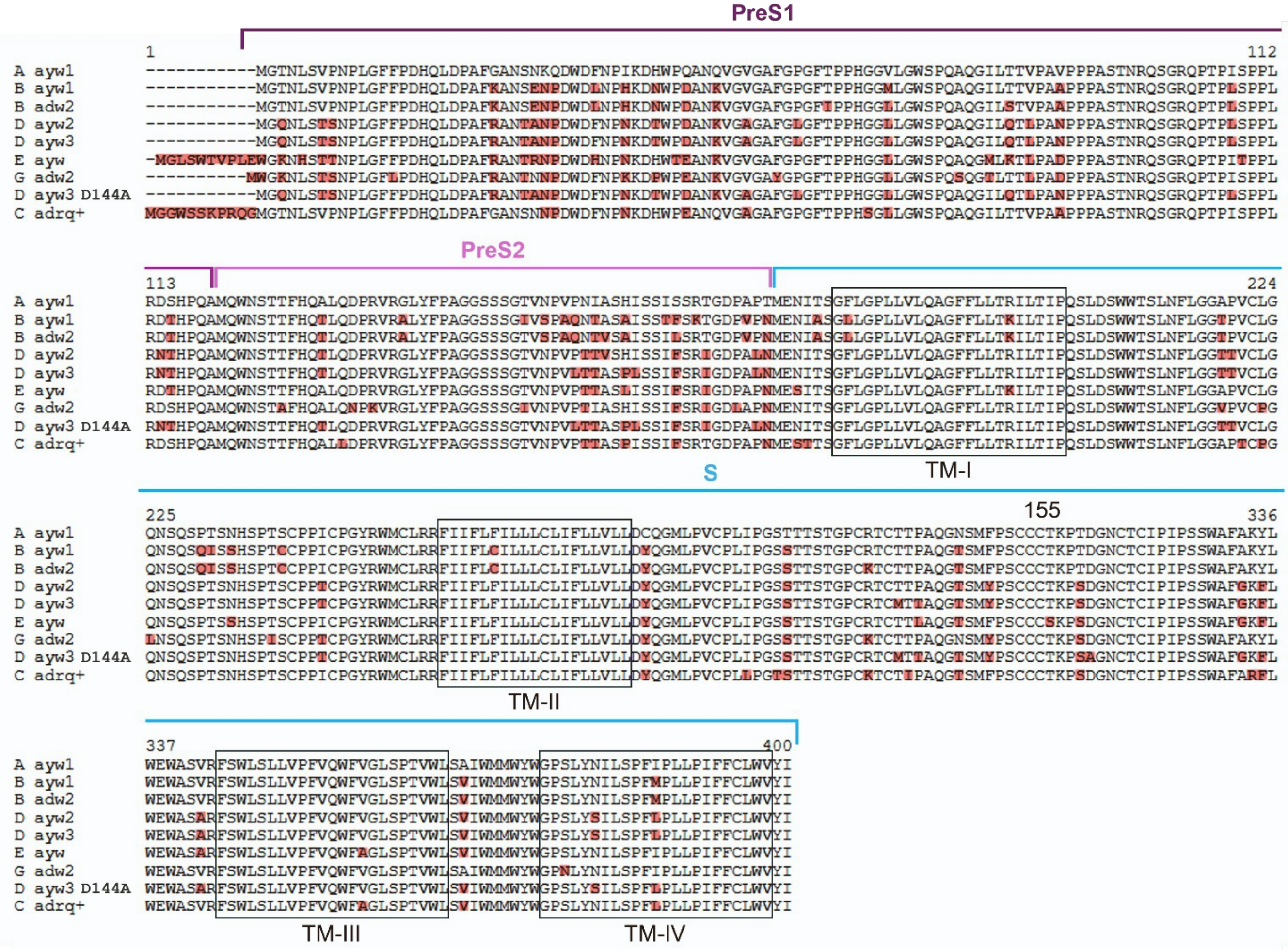

Figure S4. Alignment of L-HBsAg amino acid sequence of various HBV genotypes, related to Figure 3.

GenBank accession numbers for the sequences included in the alignment analysis are as follows: A ayw1: MW357583.1, A adw2: AY128092.1, B adw2: MW357584.1, B ayw1: MW357585.1, D ayw2: MW357587.1, D ayw3: NC_003977.2, E ayw4: MW357589.1, G adw2: MW357591.1, D ayw3 D144A: KF170746.1, C adrq+: MW357586.1. Discordant amino acids between genotypes are highlighted in red. PreS1 domain (purple), PreS2 domain (pink), and S domain (blue) are as indicated. The four transmembrane domains (TM) are framed in black[1-3].

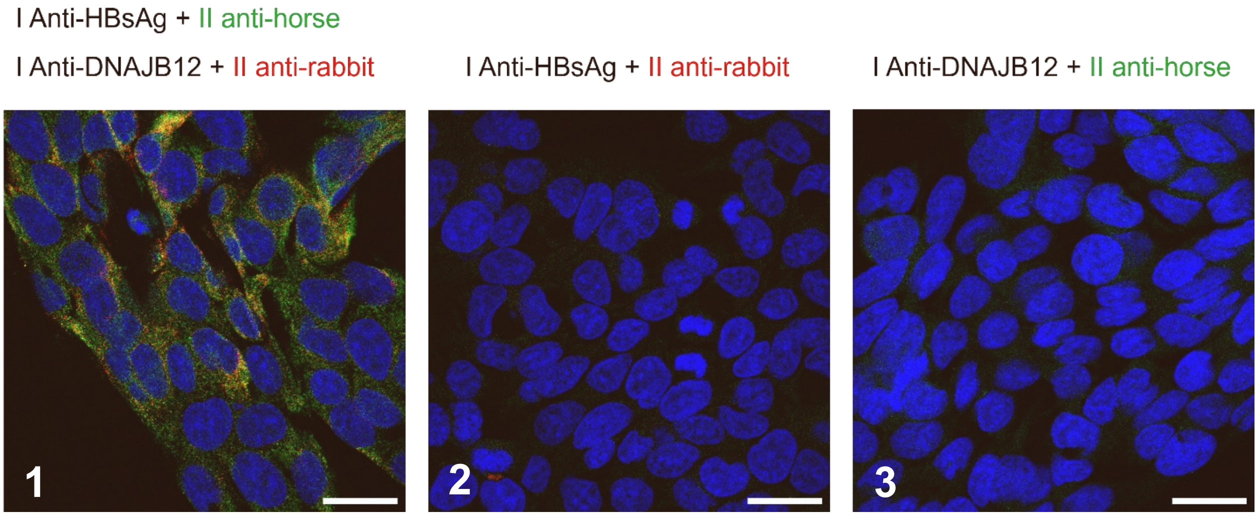

Figure S5. Cross-reactivity analysis of antibodies in immunofluorescence co-staining with anti-HBsAg and anti-DNAJB12, related to Figure 2.

To confirm the specificity of the staining observed in Figure 2, HepG2.2.15 cells were cultured, fixed, and permeabilized as described in the methods. Incubation with primary antibodies (2 h at RT) was either performed with a rabbit anti-DNAJB12 (Sigma) and a horse anti-HBsAg (Abcam) (panel 1), horse anti-HBsAg only (panel 2), or rabbit anti-DNAJB12 only (panel 3). The cells incubated with both primary antibodies were then incubated with the corresponding secondary antibodies: (1) anti-horse Alexa Fluor® 488 (green) and anti-rabbit Alexa Fluor® 568 (red) for 1 h at RT. (2) Cells incubated with horse anti-HBsAg primary antibody were then incubated with anti-rabbit Alexa Fluor® 568 (red) for 1 h at RT. (3) Cells incubated with rabbit anti-DNAJB12 primary antibody were then incubated with anti-horse Alexa Fluor® 488 (green) for 1 h at RT. Cell nuclei were stained with DAPI (blue). Cells were analyzed using a confocal microscope (Zeiss LSM 780). Scale bar: 25 µM. While we can observe a slight autofluorescence of the cells, no cross-reactivity was observed between primary and secondary antibodies.

Supplementary Table

Table S1. NAPs-interacting proteins selected for their cellular localization and functions, related to Figure 1.

Protein name	Gene name	Uniprot Entry name	log_2 (fold change) REP 2139/ REP 2179	$-log_{10}$ (p-value) REP 2139/ REP 2179	log_2 (fold change) REP 2139/ REP 2147	$-log_{10}$ (p-value) REP 2139/ REP 2147	Cellular localization	Major functions
Casein kinase I isoform delta	CSNK1D	P48730 KC1D_HUMAN	1,694	3,496	10,468	3,955	Cytoplasm and nucleus	Essential serine/threonine kinase regulating various cellular growth and survival processes.[4]
DnaJ homolog subfamily B member 12	DNAJB12	Q9NXW2 DJB12_HUMAN	1,630	3,398	4,766	3,238	Endoplasmic reticulum (ER)	Co-chaperone involved in protein folding, trafficking, and addresses unfolded protein to ER-associated degradation (ERAD) pathway.[5,6]
Coatomer subunit epsilon	COPE	O14579 COPE_HUMAN	2,525	1,326	4,574	1,303	Cytoplasm	Subunit of the coatomer multimeric complex, mediates transport between Golgi stacks and Golgi to ER.[7]
Casein kinase I isoform alpha	CSNK1A1	P48729 KC1A_HUMAN	1,936	1,601	3,673	1,952	Cytoplasm	Serine/threonine kinase, involved in several signaling pathways and diverse cellular processes.[8]
Transducin beta-like protein 2	TBL2	Q9Y4P3 TBL2_HUMAN	1,045	2,433	4,277	2,675	Endoplasmic reticulum (ER)	PERK-interacting protein, potential regulator of the PERK pathway.[9]
Microtubule-actin cross-linking factor 1	MACF1	Q9UPN3 MACF1_HUMAN	-0,066	0,028	7,225	1,852	Cytoplasm	Coordinates various cytoskeletal actions, including cell proliferation and migration.[10]
Coatomer subunit alpha	COPA	P53621 COPA_HUMAN	-0,022	0,031	3,466	2,604	Cytoplasm	Subunit of the coatomer multimeric complex, mediates transport between Golgi stacks and Golgi to ER.[7]

Supplementary References

1 Blanchet, M. & Sureau, C. Infectivity determinants of the hepatitis B virus pre-S domain are confined to the N-terminal 75 amino acid residues. *J Virol* **81**, 5841-5849 (2007). https://doi.org:10.1128/jvi.00096-07

2 Hartmann-Stühler, C. & Prange, R. Hepatitis B Virus Large Envelope Protein Interacts with γ2-Adaptin, a Clathrin Adaptor-Related Protein. *Journal of Virology* **75**, 5343-5351 (2001). https://doi.org:doi:10.1128/jvi.75.11.5343-5351.2001

3 Rezaee, R. *et al.* Impacts of the G145R Mutation on the Structure and Immunogenic Activity of the Hepatitis B Surface Antigen: A Computational Analysis. *Hepat Mon* **16**, e39097 (2016). https://doi.org:10.5812/hepatmon.39097

4 Xu, P. *et al.* Structure, regulation, and (patho-)physiological functions of the stress-induced protein kinase CK1 delta (CSNK1D). *Gene* **715**, 144005 (2019). https://doi.org:10.1016/j.gene.2019.144005

5 Yamamoto, Y. H. *et al.* A novel ER J-protein DNAJB12 accelerates ER-associated degradation of membrane proteins including CFTR. *Cell Struct Funct* **35**, 107-116 (2010). https://doi.org:10.1247/csf.10023

6 Grove, D. E., Fan, C. Y., Ren, H. Y. & Cyr, D. M. The endoplasmic reticulum-associated Hsp40 DNAJB12 and Hsc70 cooperate to facilitate RMA1 E3-dependent degradation of nascent CFTRDeltaF508. *Mol Biol Cell* **22**, 301-314 (2011). https://doi.org:10.1091/mbc.E10-09-0760

7 Taylor, R. J., Tagiltsev, G. & Briggs, J. A. G. The structure of COPI vesicles and regulation of vesicle turnover. *FEBS Lett* **597**, 819-835 (2023). https://doi.org:10.1002/1873-3468.14560

8 Spinello, Z. *et al.* Targeting Protein Kinases in Blood Cancer: Focusing on CK1α and CK2. *International Journal of Molecular Sciences* **22**, 3716 (2021).

9 Tsukumo, Y. *et al.* TBL2 is a novel PERK-binding protein that modulates stress-signaling and cell survival during endoplasmic reticulum stress. *PLoS One* **9**, e112761 (2014). https://doi.org:10.1371/journal.pone.0112761

10 Miao, Z. *et al.* Microtubule actin cross-linking factor 1, a novel potential target in cancer. *Cancer Sci* **108**, 1953-1958 (2017). https://doi.org:10.1111/cas.13344

Data S1: Raw images of the Western Blots

Prestained protein standards used are either the Precision Plus Protein™ Kaleidoscope™ Prestained Protein Standards (Bio-Rad) or the Precision Plus Protein™ Dual Color Standards (Bio-rad).

In some pictures, only a small portion of the WB was used in the figure. In these cases, a red rectangle was added to help localise the relevant bands.

Figure 1

Figure 1G:

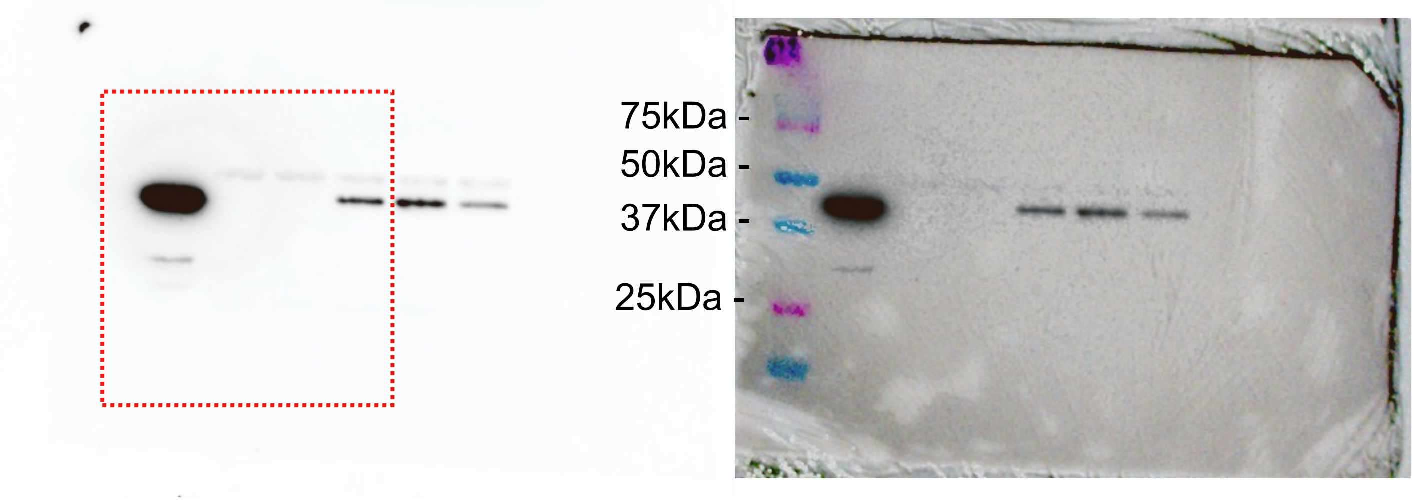

Figure 1H:

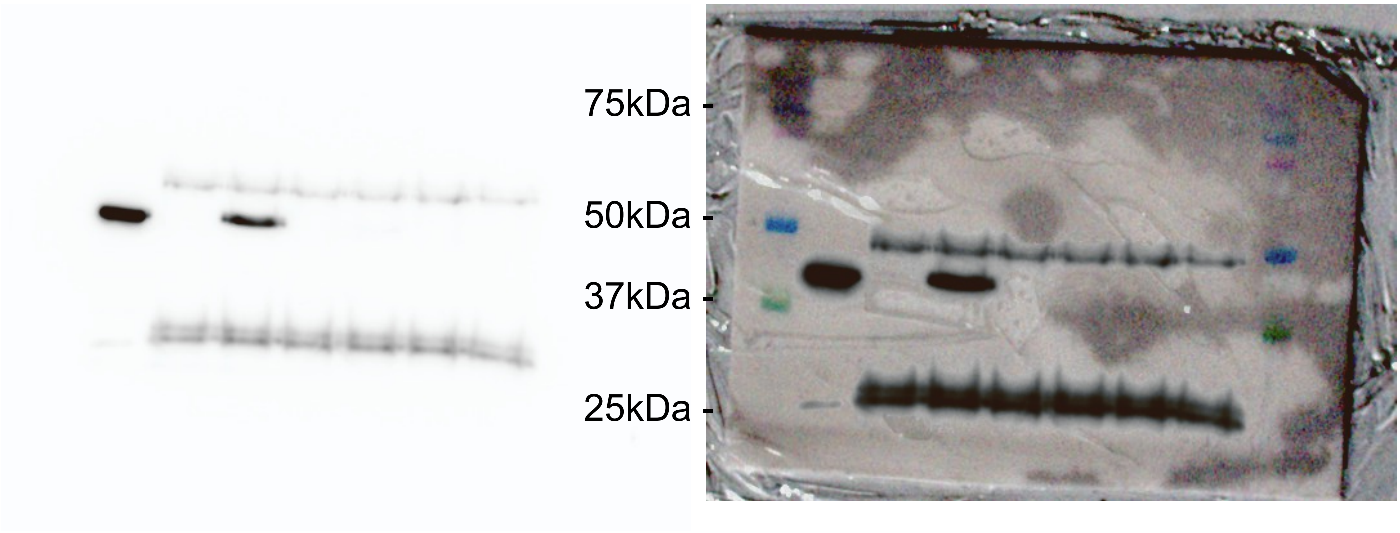

Figure 2

Figure 2B:

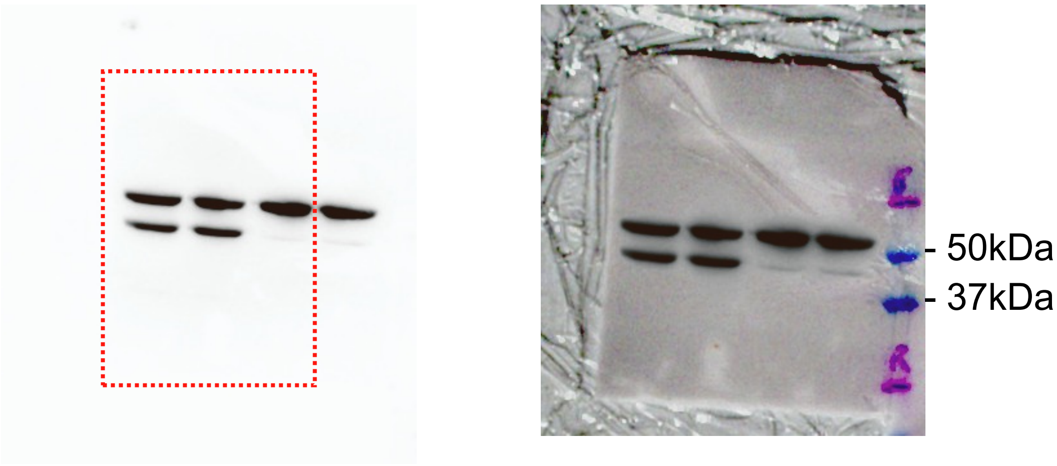

Figure 3

Figure 3C, upper panel:

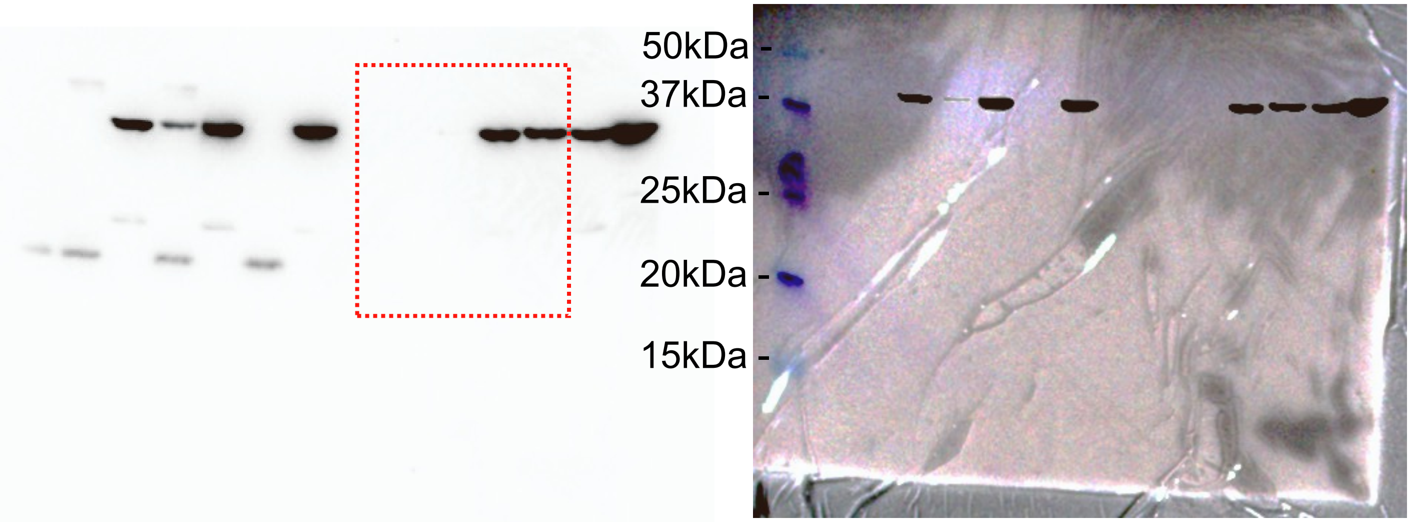

Figure 3C, lower panel:

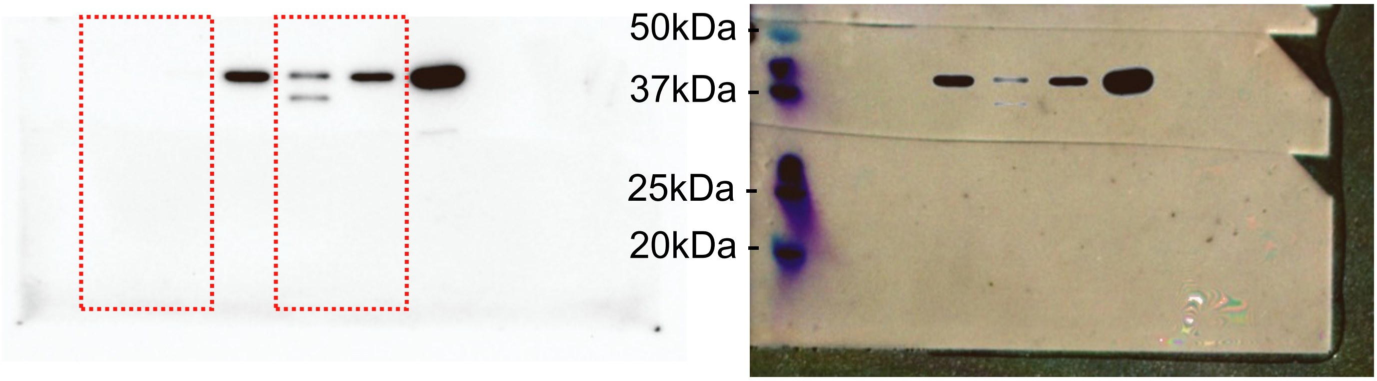

Figure 3

Figure 3D, upper panel:

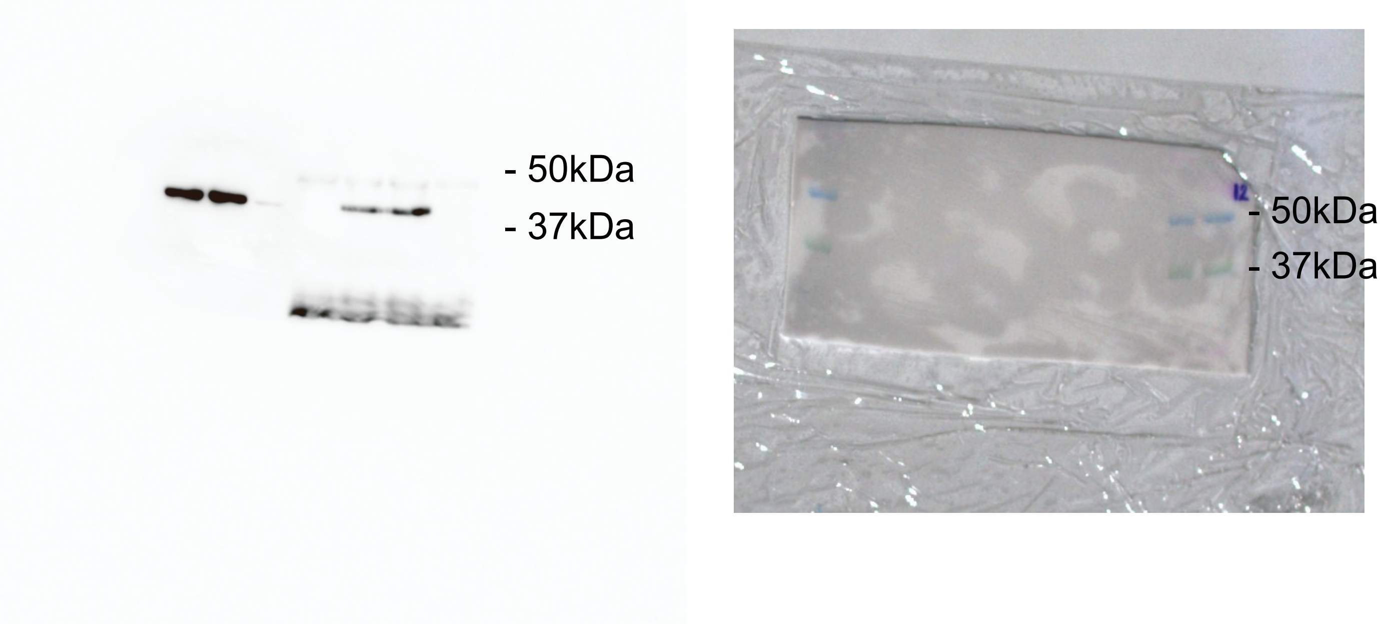

Figure 3D, lower panel:

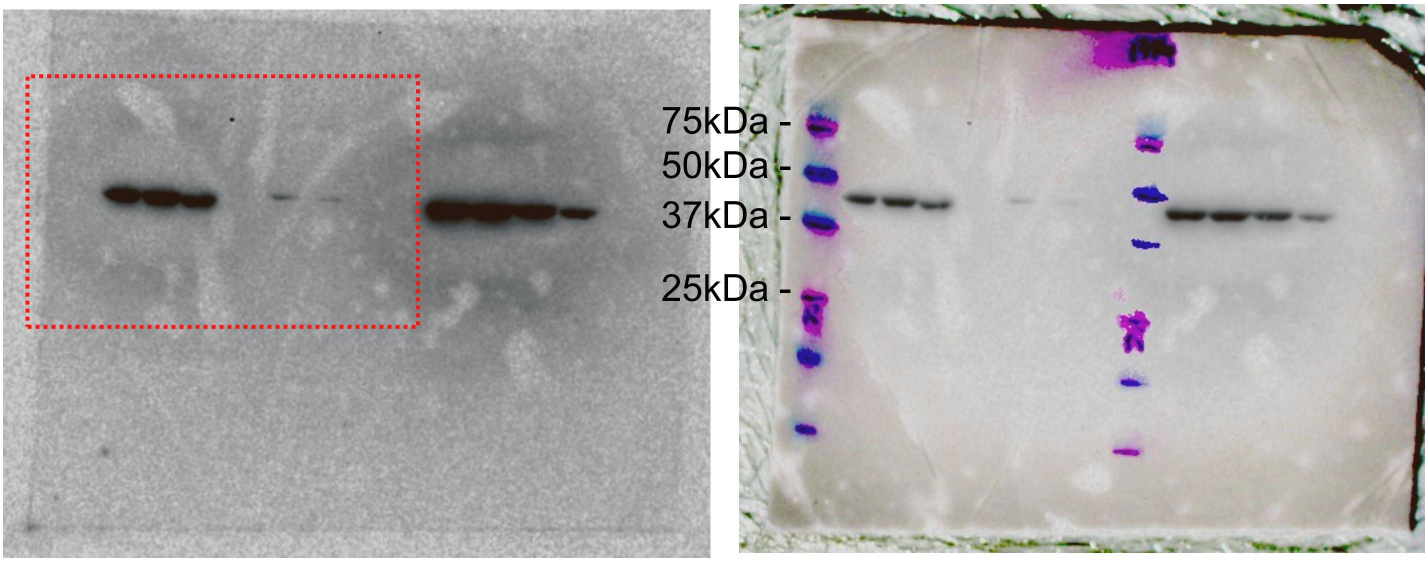

Figure 3

Figure 3E, left panels:

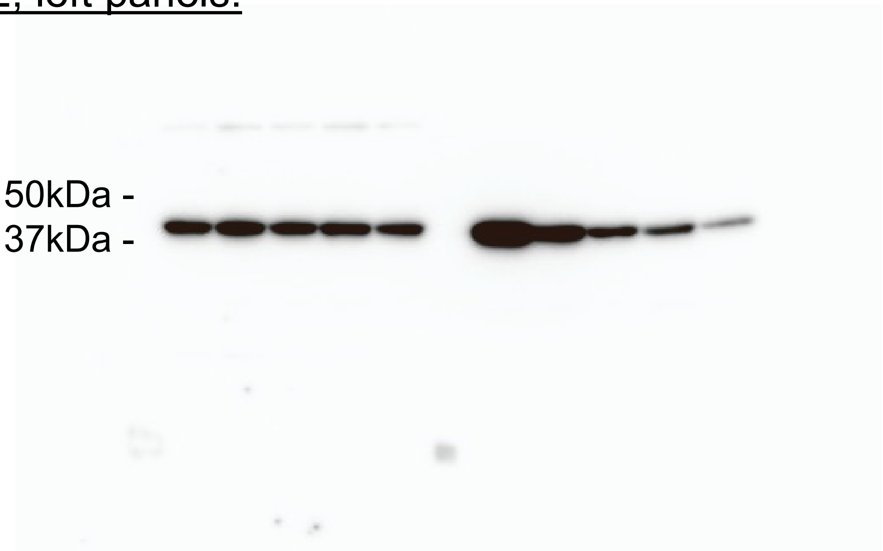

Figure 3E, right panels:

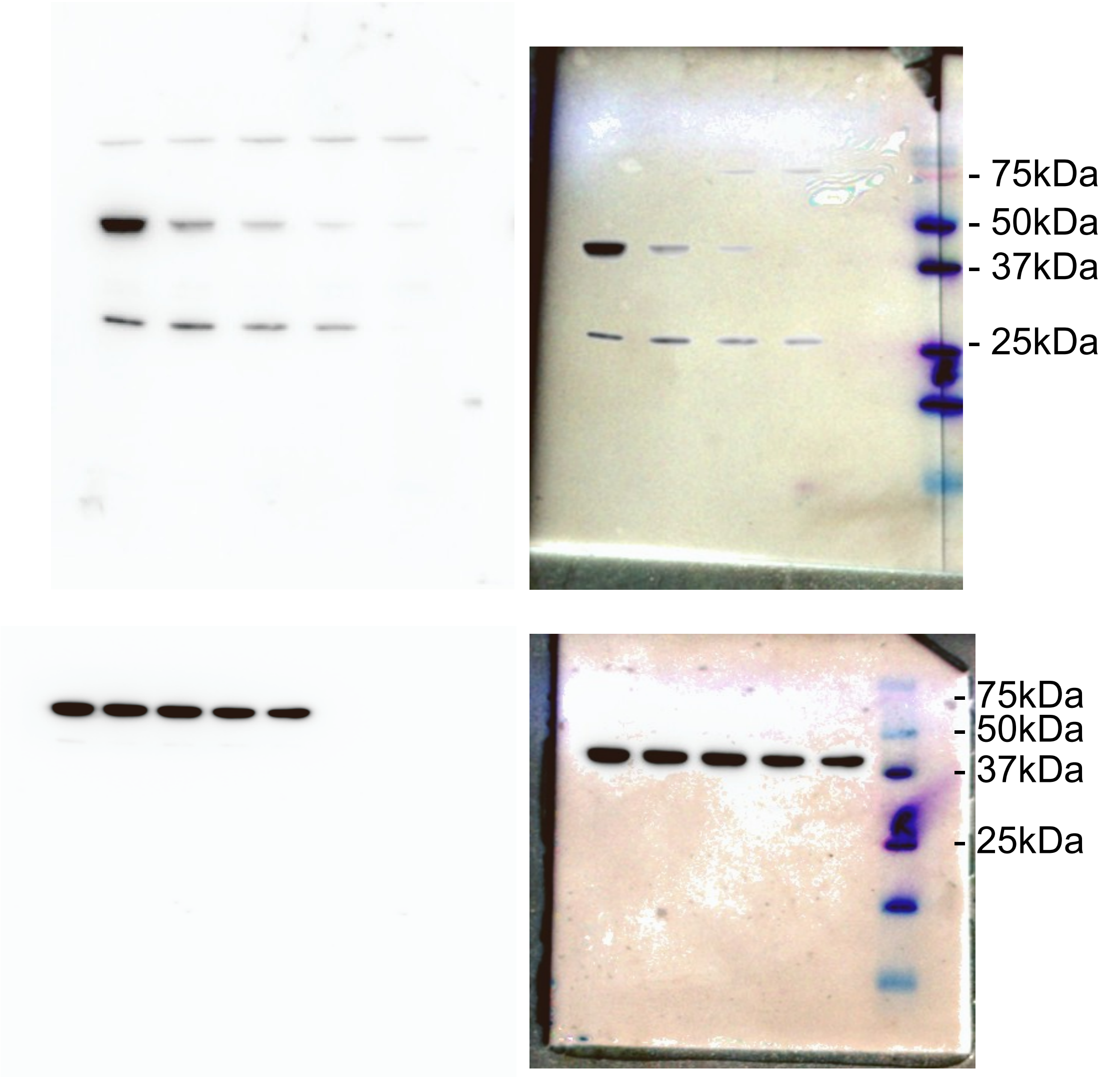

Figure 3

Figure 3H, upper panels:

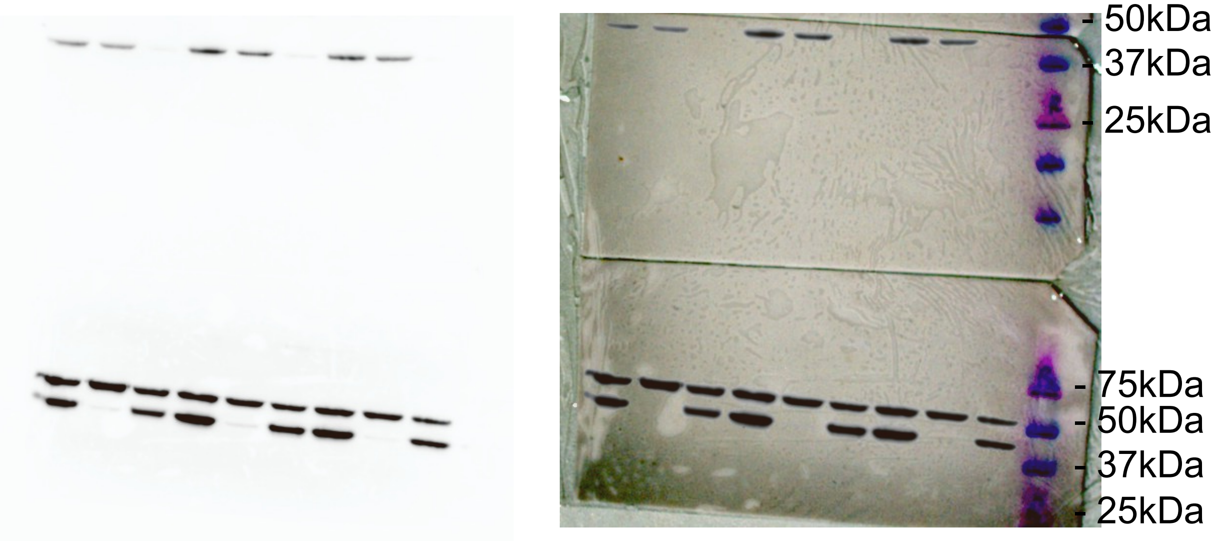

Figure 3H, mid panels:

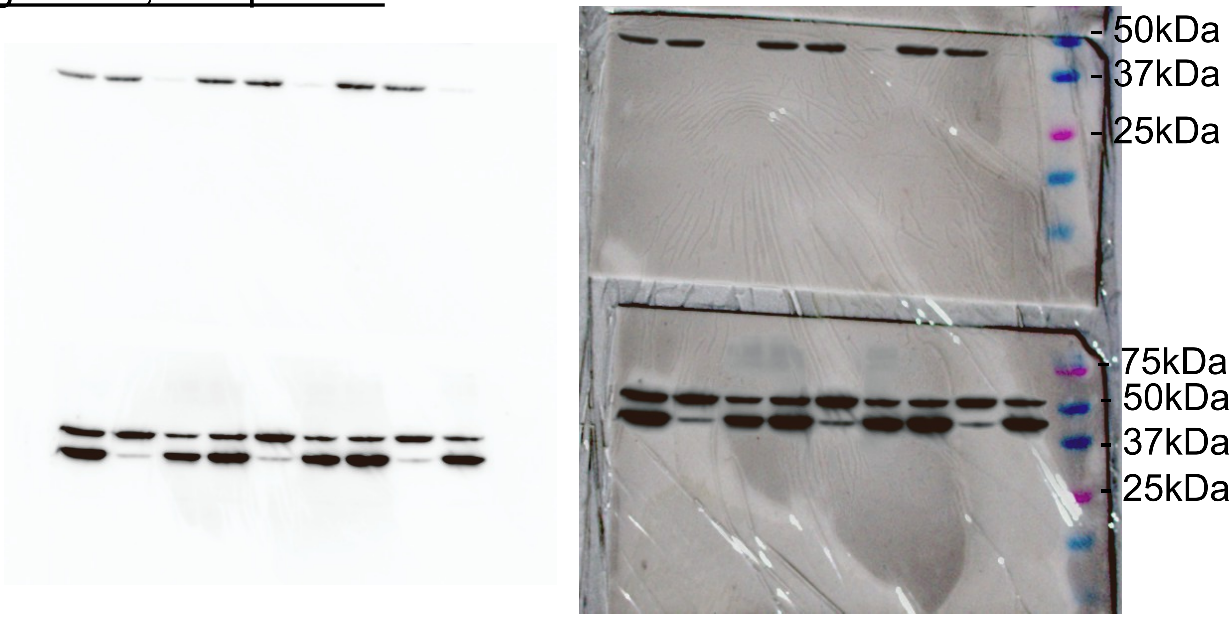

Figure 3H, lower panels:

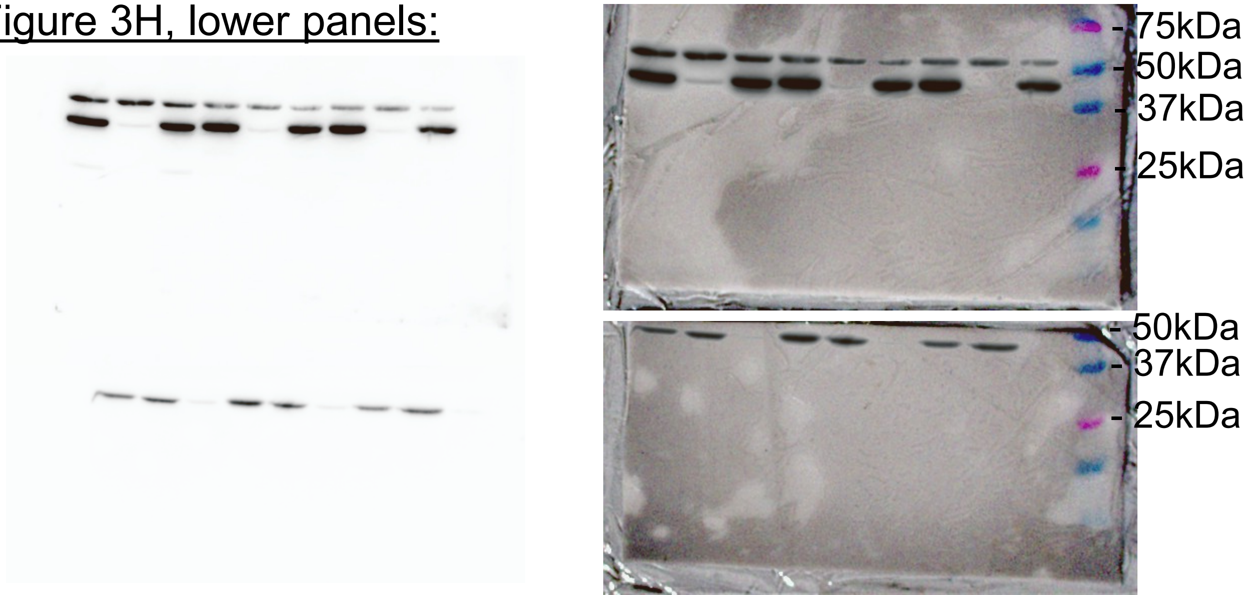

Figure 4

Figure 4A:

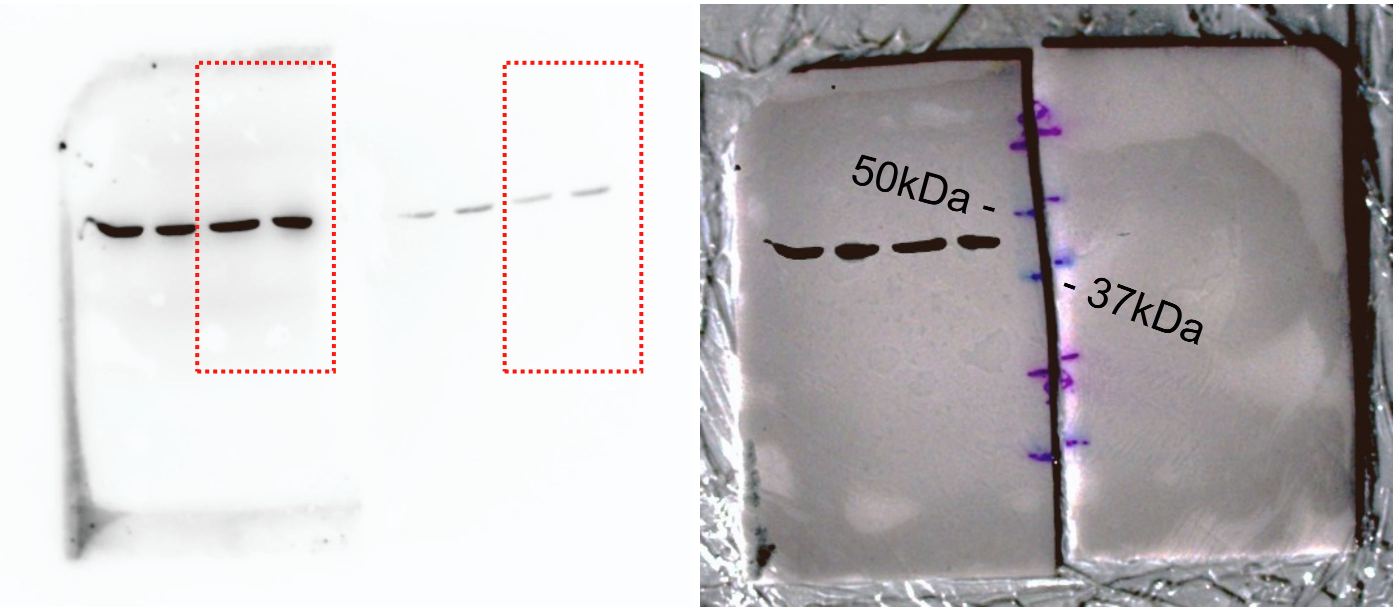

Figure 5

Figure 5C:

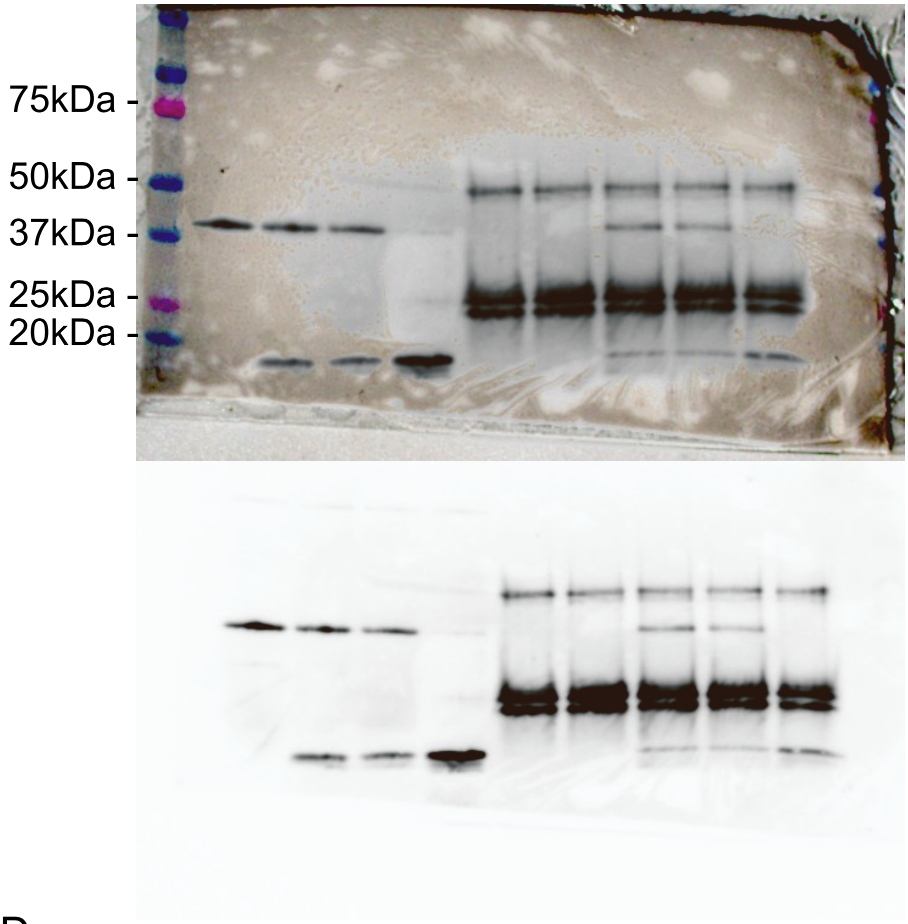

Figure 5D:

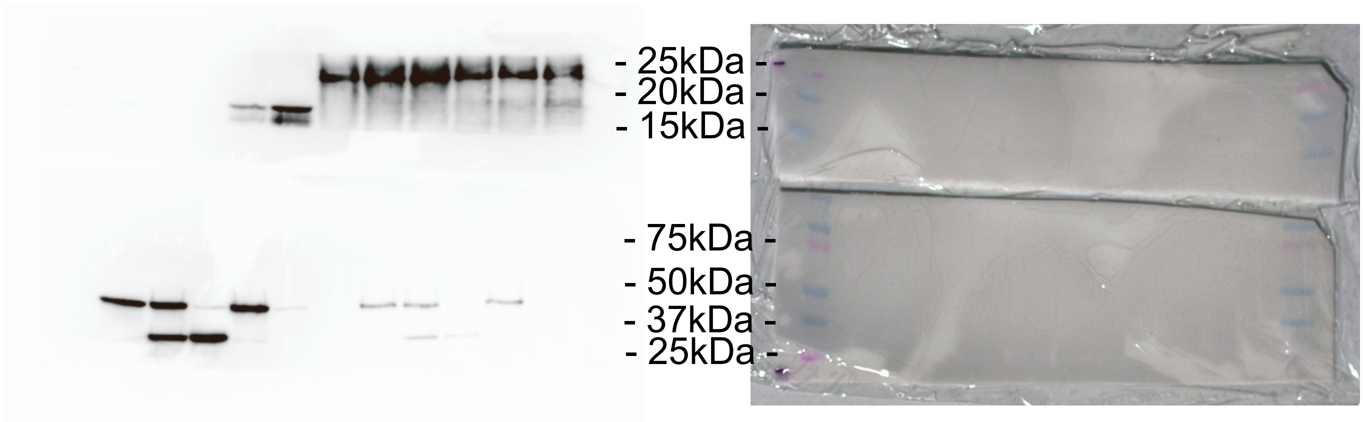

Figure 6

Figure 6B, upper panels:

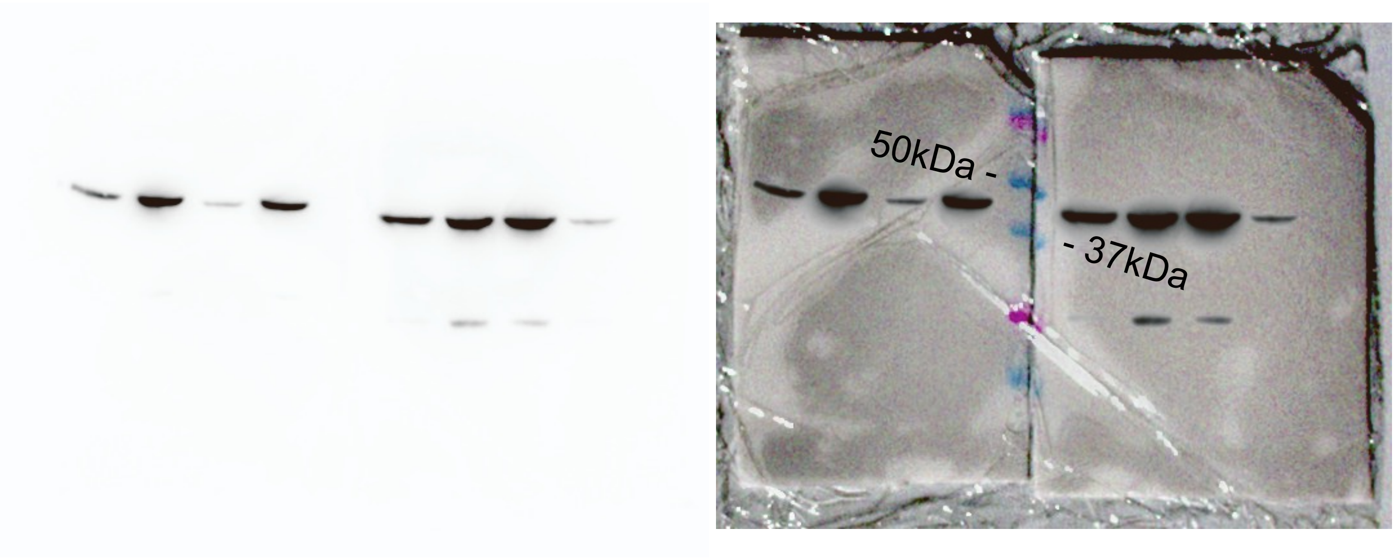

Figure 6B, lower panels:

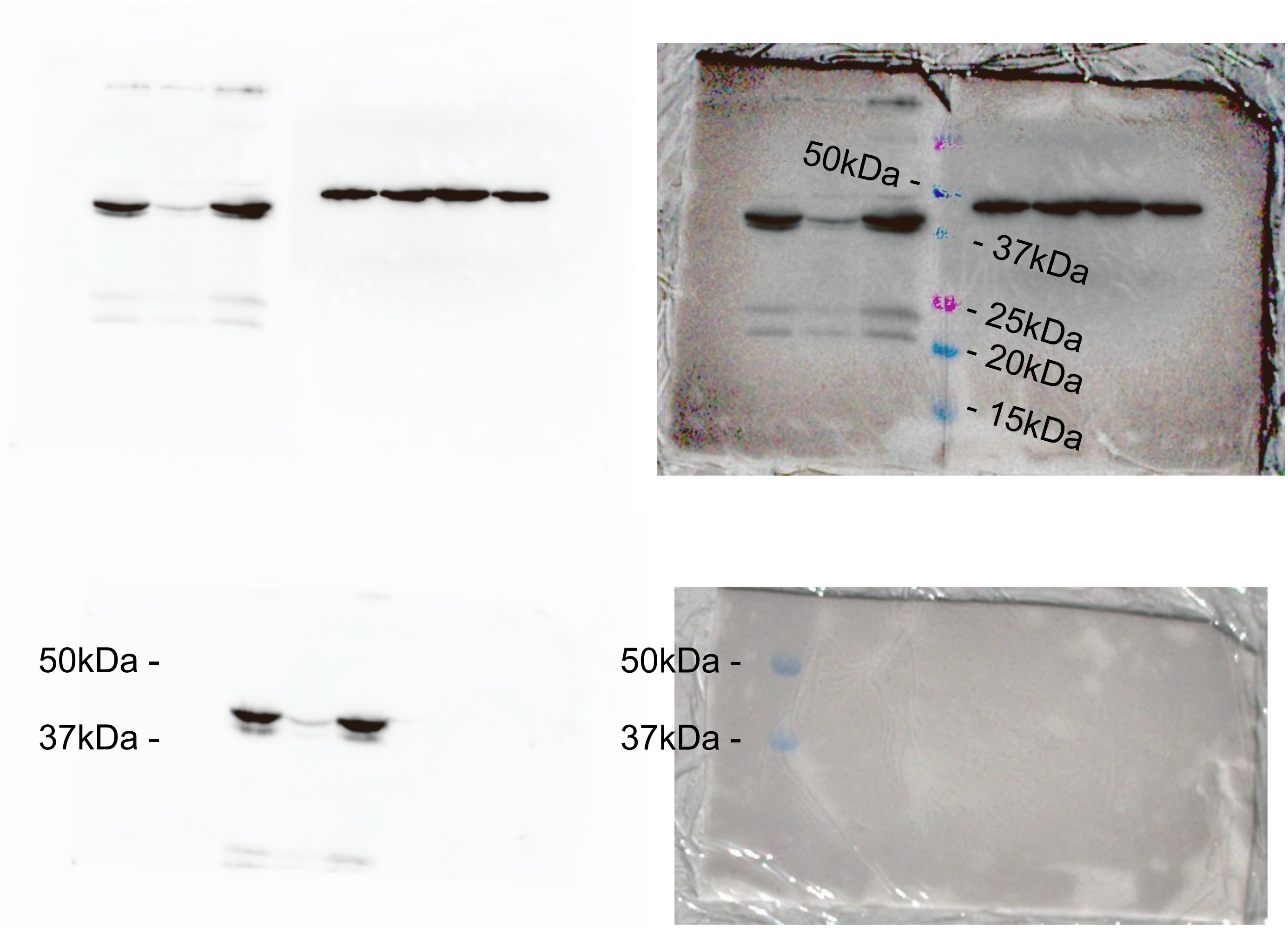

Supplement: Document S1. Figures S1–S5, Table S1, and Data S1 [file mmc1.pdf]
